# Structure Elucidation and Biosynthesis of Nannosterols A and B, Myxobacterial Sterols from *Nannocystis* sp. MNa10993

**DOI:** 10.1021/acs.jnatprod.2c01143

**Published:** 2023-04-03

**Authors:** Sergi H. Akone, Joachim J. Hug, Amninder Kaur, Ronald Garcia, Rolf Müller

**Affiliations:** † Helmholtz-Institute for Pharmaceutical Research Saarland (HIPS), Helmholtz Centre for Infection Research (HZI), Department of Microbial Natural Products, Saarland University, Campus E8 1, 66123 Saarbrücken, Germany; ‡ Department of Pharmacy, Saarland University, Campus E8 1, 66123 Saarbrücken, Germany; § German Center for Infection Research (DZIF), Partner Site Hannover-Braunschweig, 38124 Braunschweig, Germany; ⊥ Helmholtz International Laboratories, Department of Microbial Natural Products, Saarland University, Campus E8 1, 66123 Saarbrücken, Germany; ∥ Department of Chemistry, Faculty of Science, University of Douala, P.O. Box 24157, Douala, Cameroon

## Abstract

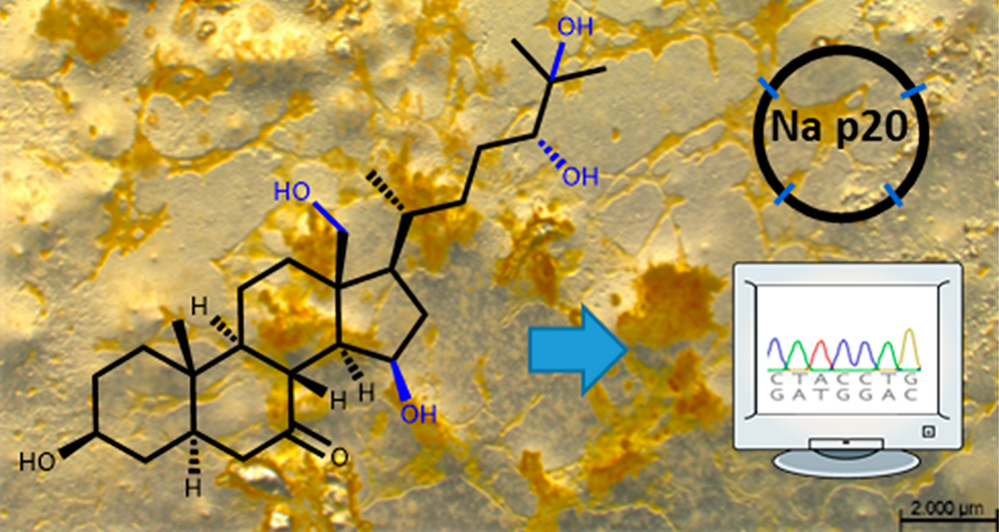

Myxobacteria represent an underinvestigated source of chemically diverse and biologically active secondary metabolites. Here, we report the discovery, isolation, structure elucidation, and biological evaluation of two new bacterial sterols, termed nannosterols A and B (**1**, **2**), from the terrestrial myxobacterium *Nannocystis* sp. (MNa10993). Nannosterols feature a cholestanol core with numerous modifications including a secondary alcohol at position C-15, a terminal vicinal diol side chain at C-24–C-25 (**1**, **2**), and a hydroxy group at the angular methyl group at C-18 (**2**), which is unprecedented for bacterial sterols. Another rare chemical feature of bacterial triterpenoids is a ketone group at position C-7, which is also displayed by **1** and **2**. The combined exploration based on myxobacterial high-resolution secondary metabolome data and genomic *in silico* investigations exposed the nannosterols as frequently produced sterols within the myxobacterial suborder of Nannocystineae. The discovery of the nannosterols provides insights into the biosynthesis of these new myxobacterial sterols, with implications in understanding the evolution of sterol production by prokaryotes.

Myxobacteria provide an underexploited reservoir of structurally unique natural products featuring interesting biological functions.^1^ The occupied chemical space of bacterial natural products differs significantly from plants and fungi,^2,3^ as exemplified by hybridized chemical scaffolds of myxobacterial natural products originating from the linkage of carboxylic acids and amino acids.^4,5^

The majority of these complex natural products are generated by enzyme complexes of the multimodular nonribosomal peptide synthetase (NRPS) and polyketide synthase (PKS) types, and the combinatorial nature of these megasynthases is partly responsible for the impressive structural diversity of myxobacterial natural products.^6^ Additionally, bacteria rarely produce cholesterol-derived steroids, which are commonly encountered from eukaryotic organisms, as these assist in controlling the fluidity and flexibility of their cell membranes^7^ and work as signaling molecules.^8^ In recent years, an increasing number of laboratories have dissected the differences between prokaryotic and eukaryotic triterpene biogenesis, thereby providing unique evolutionary insights.^9,10^

Herein we report the isolation, structure elucidation, and biological evaluation of two new bacterial sterols named nannosterols A and B (**1**, **2**) from a novel myxobacterial isolate, *Nannocystis* sp. (MNa10993) (Figure 1). Furthermore, *in silico* biosynthetic investigation combined with metabolome data evaluation supports the notion that the nannosterols are frequently produced sterols within the myxobacterial suborder of Nannocystineae and might provide another puzzle piece to reveal the enigmatic nature of bacterial sterol biosynthesis.

**Figure 1:**
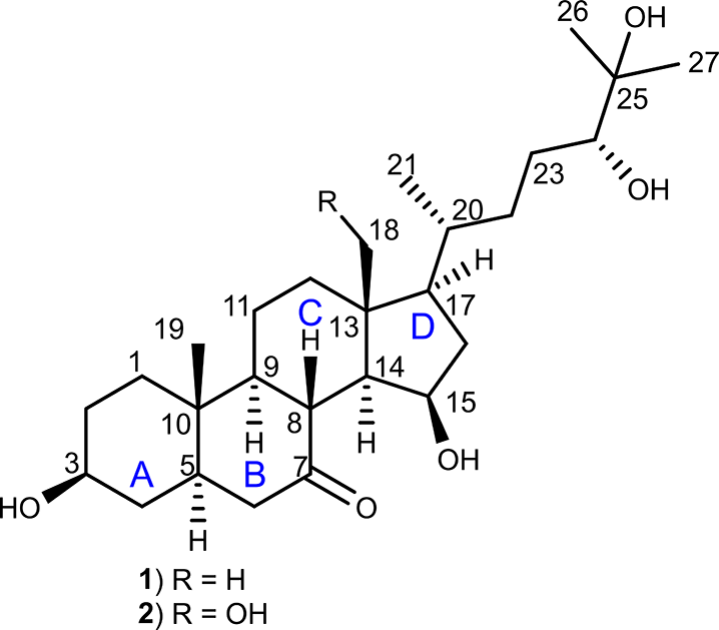


## Results and Discussion

### Discovery, Isolation, and Structure Elucidation of Nannosterols

**Figure 2:**
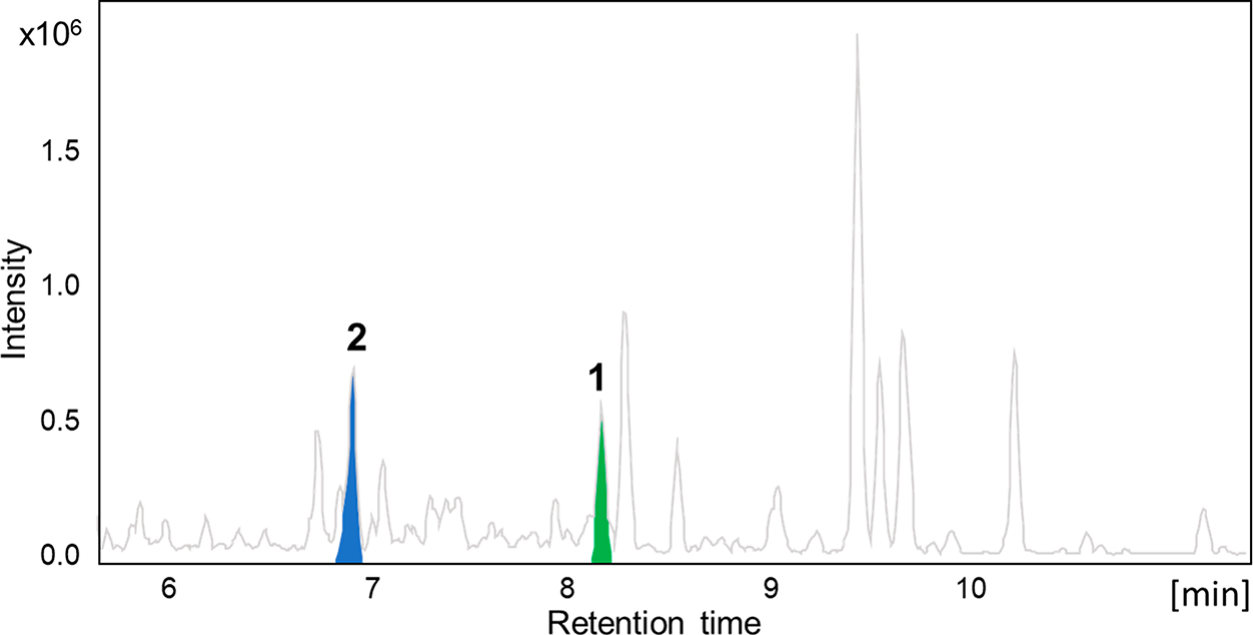
High-performance liquid chromatography–mass spectrometry base peak chromatogram (HPLC-MS BPC) (gray) and extracted ion chromatogram (EIC) of MNa10993 extracts, displaying the peaks of **1** ([M + H]^+^ 451.3397 *m*/*z*, green) and **2** ([M + H]^+^ 467.3341 *m*/*z*, blue).

An initial high-performance liquid chromatography–mass spectrometry (HPLC-MS) analysis of the secondary metabolome of the crude extract of *Nannocystis* sp. (MNa10993) and comparison of this data to that included in the HPLC-MS metabolome database named Myxobase^11^ enabled the identification of two unknown secondary metabolites (Figure 2).

Target metabolites with masses corresponding to 467.3357 [M + H]^+^ and 451.3421 [M + H]^+^ were detected at retention times of 6.93 and 8.53 min. An initial literature search using the molecular formulas of **1** and **2** yielded numerous compounds of the cholestane family, but their correct identification could only be achieved by complete structure elucidation using 1D and 2D NMR data. Considering that bacteria rarely synthesize steroids, the methanol extract of MNa10993 was further investigated. Large-scale fermentation of *Nannocystis* sp. MNa10993 permitted the isolation of **1** and **2** in sufficient amounts for full structure elucidation.

**Figure 3:**
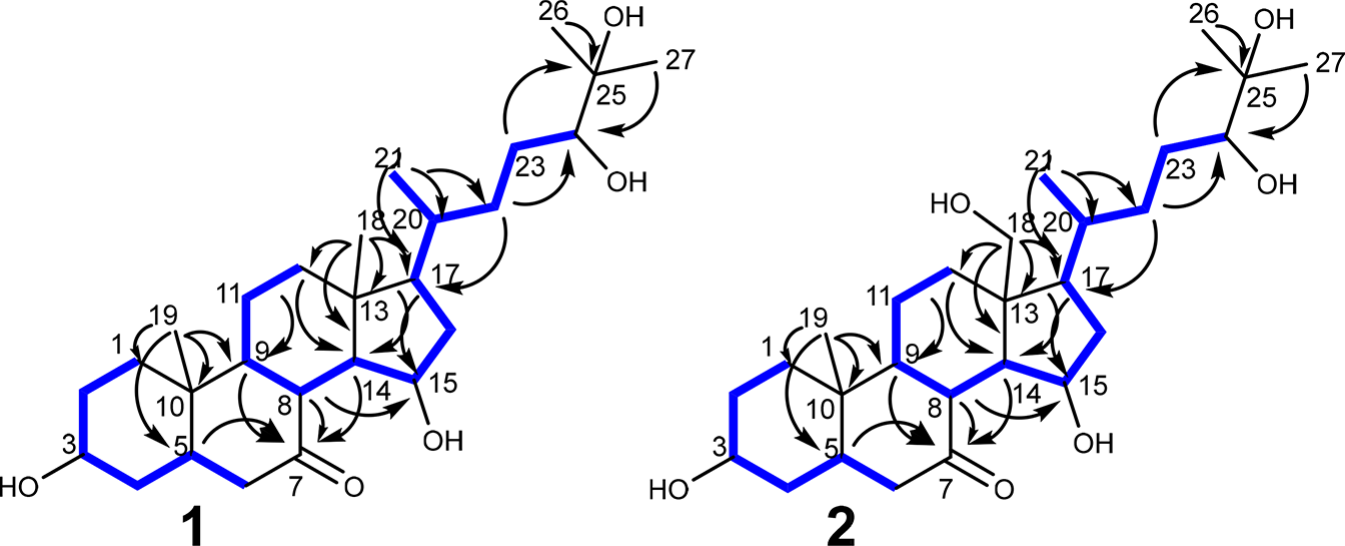
Key COSY (blue bold) and HMBC (arrows) correlations of **1** and **2**.

Compound **1** was obtained as an amorphous white powder. Its molecular formula was established as C_27_H_46_O_5_ based on the pseudomolecular ion peak at *m*/*z* 451.3421 in the high-resolution electrospray ionization mass spectrometry (HRESIMS) spectrum, which requires five degrees of unsaturation. The ^1^H NMR and edited HSQC spectra of **1** (Table 1) displayed signals attributed to nine methines, three of which were oxygenated at δ_H_ 3.54 (m, H-3), 4.64 (m, H-15), and 3.22 (br d, *J* = 9.8 Hz, H-24), nine methylenes, and five methyl groups including one methyl doublet at δ_H_ 0.96 (d, *J* = 6.5 Hz, H_3_-21) and four singlets at δ_H_ 0.93 (s, H_3_-18), 1.14 (s, H_3_-19), 1.13 (s, H_3_-26), and 1.16 (s, H_3_-27), corresponding to tertiary methyl groups. In light of the molecular formula, the remaining four hydrogens are due to the presence of four exchangeable hydroxy groups in the molecule at δ_H_ 4.54 (br d, *J* = 4.9 Hz, 3-OH), 3.92 (br d, *J* = 2.9 Hz; 15-OH), 4.18 (br d, *J* = 6.0 Hz; 24-OH), and 4.01 (br s, 25-OH). Fully decoupled ^13^C and DEPT-135 NMR spectra of **1** revealed 27 well-resolved carbon resonances (Table 1). In addition to the aforementioned proton-bearing carbons, one ketocarbonyl at δ_C_ 214.6 and two quaternary carbons were recognized, which was accounted for by one degree of double-bond equivalents. The remaining four sites of unsaturation suggested that **1** was a tetracyclic compound. The spin system from H_2_-1 to H_2_-6, as well as the vicinal coupling of H_2_-9/H_2_-8 displayed on the ^1^H–^1^H COSY spectrum of **1**, along with the HMBC correlations from H_3_-19 to C-1 (δ_C_ 37.0), C-5 (δ_C_ 48.2), C-9 (δ_C_ 56.9), and C-10 (δ_C_ 37.1) and from H-5 and H-9 to C-7 (*δ*_*C*_ 214.6), generated rings A and B with an angular methyl at C-10 (Figure 3), which also proved the unusual presence of a ketone at C-7. Further analysis of the ^1^H–^1^H COSY spectrum of **1** allowed the identification of an additional spin system from H-12 to H_2_-17. The latter COSY correlations together with the HMBC correlations from H_3_-18 to C-12 (δ_C_ 41.2), C-13 (δ_C_ 43.2), C-14 (δ_C_ 55.5), and C-17 (δ_C_ 56.7) corroborated the presence of rings C and D with another angular methyl group at C-13 (Figure 3). Fusion of ring C and D was based on the HMBC correlations of H-8, H-9, and H-14 to C-7 (δ_C_ 214.6) (Figure 3). The presence of a hydroxy group at C-15 was supported by the HMBC correlation from H-17 and H-8 to C-15, along with the characteristic chemical shift (δ_H_ 4.64, δ_C_ 70.8). Moreover, the ^1^H–^1^H COSY correlations from the H_2_-17, via H_3_-21, to H-24 along with the HMBC correlations from H_3_-26, H_3_-27, and H_2_-23 to C-24 (δ_C_ 79.8) and C-25 (δ_C_ 73.9) constructed the steroidal side chain of 2,6-dimethylhexane-2,3-diol (Figure 3). The latter was further confirmed by the HMBC cross-peaks from H_3_-21 to C-20 (δ_C_ 36.3), C-17 (δ_C_ 56.7), and C-22 (δ_C_ 34.1) (Figure 3). The aforementioned pattern is commonly observed in compounds bearing a cholestane moiety. Notably, hydroxy groups at positions C-24, C-25, and C-15 are not commonly encountered in the cholestanes produced by microorganisms.

**Table 1:** NMR Data of **1** and **2** Measured in CD_3_OD at 700 (^1^H) and 175 (^13^C) MHz

	**1**		**2**	
no.	δ_C_, type	δ_H_, mult. (*J* in Hz)	δ_C_, type	δ_H_, mult. (*J* in Hz)
1	37.0, CH_2_	1.05, m	37.3, CH_2_	1.05, m
		1.78, br dt, (3.8, 13.9)		1.76, br dt, (3.4, 13.7)
2	31.7, CH_2_	1.48, m	31.9, CH_2_	1.48, m
		1.81, br d (12.3)		1.82, br d (12.2)
3	71.3, CH	3.54, m	71.5, CH	3.54, m
4	38.7, CH_2_	1.44, m	38.9, CH_2_	1.46, m
		1.58, m		1.58, m
5	48.2, CH	1.51, m	48.6, CH	1.52, m
6	46.6, CH_2_	1.97, dd (3.8, 12.7)	47.1, CH_2_	1.97, dd (2.8, 12.4)
		2.52, t (12.7)		2.53, t (12.4)
7	214.6, C		214.2, C	
8	46.8, CH	2.83, t (11.4)	46.5, CH	2.87, t (11.9)
9	56.9, CH	1.11, m	57.0, CH	1.15, m
10	37.1, C		37.5, C	
11	22.8, CH_2_	1.58, m (2H)	23.4, CH_2_	1.51, m
				1.60, m
12	41.2, CH_2_	1.09, m	37.9, CH_2_	1.05, m
		1.97, dd (3.2, 12.7)		2.27, dt (12.7, 3.4)
13	43.2, C		47.9, C	
14	55.5, CH	1.28 (dd, 5.5, 11.4)	55.2, CH	1.46, m
15	70.8, CH	4.64, m	70.5, CH	4.67, m
16	41.4, CH_2_	1.35, m	42.2, CH_2_	1.47, m
		2.44, m		2.41, m
17	56.7, CH	1.11, m	57.0, CH	1.17, m
18	14.7, CH_3_	0.93, s	62.1, CH_2_	3.63, d (12.2)
				3.85, d (12.2)
19	11.9, CH_3_	1.14, s	12.1, CH_3_	1.14, s
20	36.3, CH	1.57, m	35.9, CH	1.89, m
21	19.0, CH_3_	0.96, d (6.5)	20.0, CH_3_	1.04, s
22	34.1, CH_2_	1.35, m	34.5, CH_2_	1.33, m
		1.49, m		1.51, m
23	28.5, CH_2_	1.34, m	28.6, CH_2_	1.33, m
		1.51, m		1.52, m
24	79.8, CH	3.22, br d (9.8)	79.9, CH	3.23, br d (9.8)
25	73.9, C		74.0, C	
26	24.8, CH_3_	1.13, s	25.0, CH_3_	1.13, s
27	25.7, CH_3_	1.16, s	25.9, CH_3_	1.16, s
3-OH^a^		4.54, br d (4.9)		4.54, br d (4.9)
15-OH^a^		3.92, br d (2.9)		3.89, br d (2.9)
24-OH^a^		4.18, br d (6.0)		4.16, br s
25-OH^a^		4.01, br s		4.00, br s

a Data extracted from DMSO-*d*_6_ measurement.

**Figure 4:**
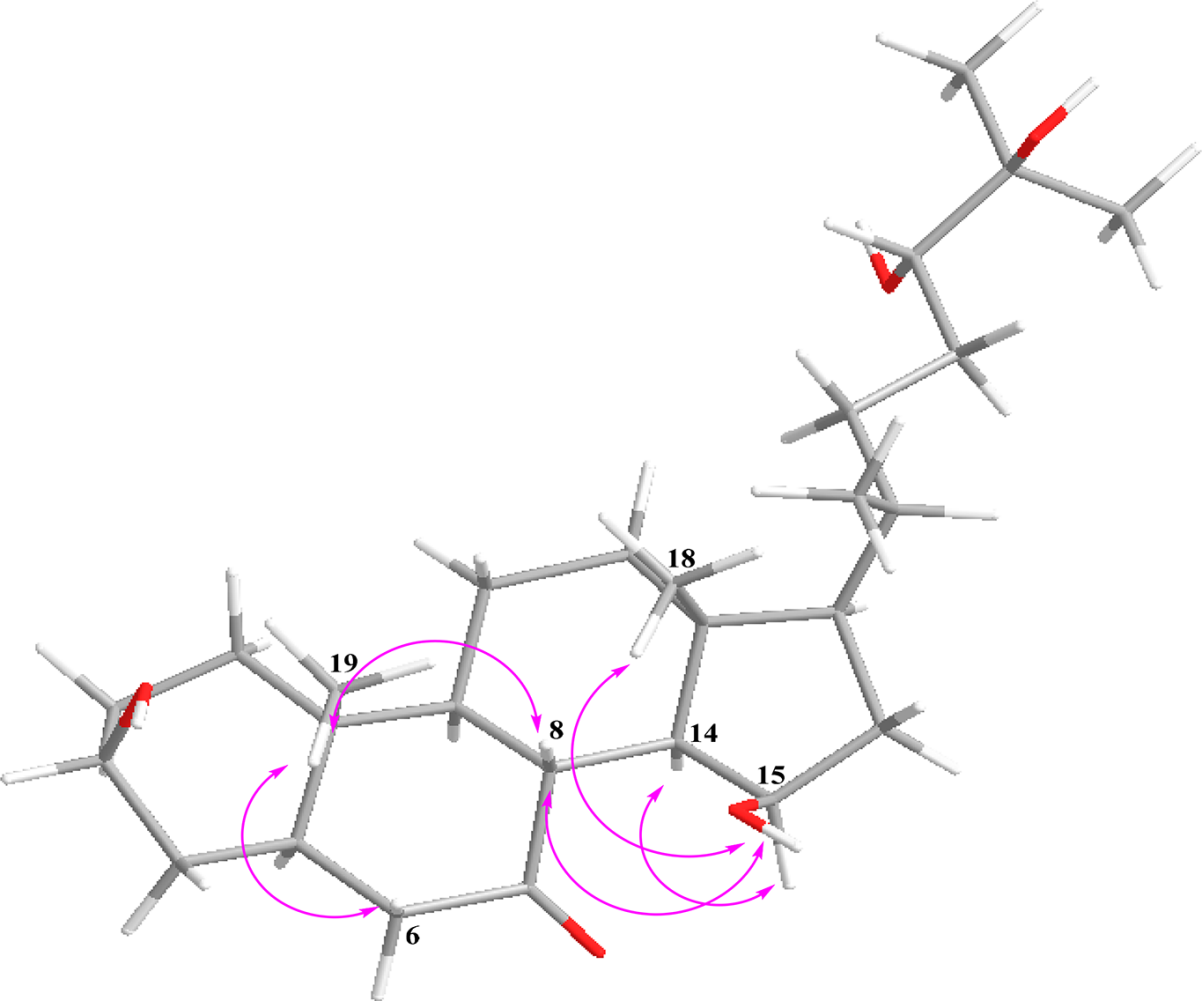
Key ROESY correlations of **1** and **2**.

A ROESY experiment in DMSO-*d*_6_ (Figure 4) was conducted to disclose the relative configuration of **1**. Key ROESY correlations were observed between H_3_-19, H-6, H-8, H_3_-18, and OH-15, as well as between H-14 and H-15, placing these *syn* to each other. The multiplicity as well as the large coupling constant between H-14 and H-8 (*J*_8–14_ = 11.4 Hz) indicated their *trans*-diaxial orientation. Overlapping signals and the lack of diagnostic correlations did not allow the assignment of the relative configuration of H-3 and H-17 of the ring system by ROESY. Additionally, the relative configuration of the stereocenters on the side chain with respect to those on the ring could not be simply assigned by ROESY NMR data due to free rotation around the C-17–C-20 single bond.

**Figure 5:**
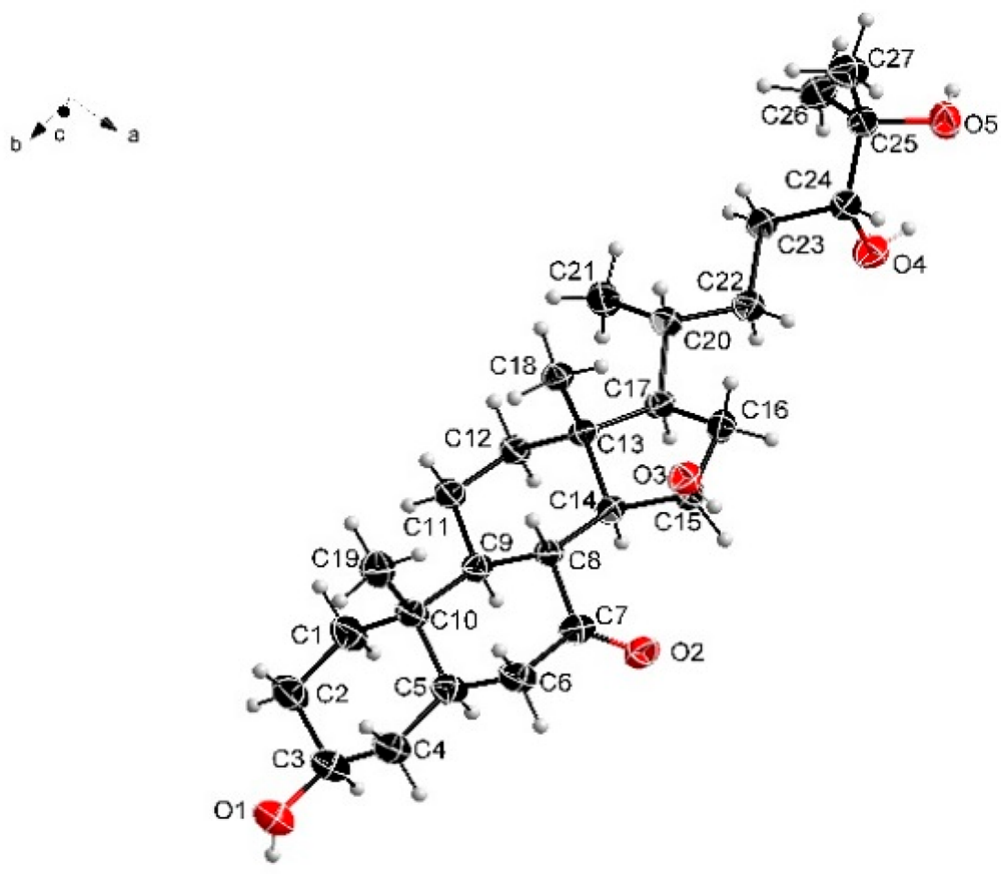
Molecular structure of **1** in the crystal (thermal ellipsoids at the 50% probability level).

**Figure 6:**
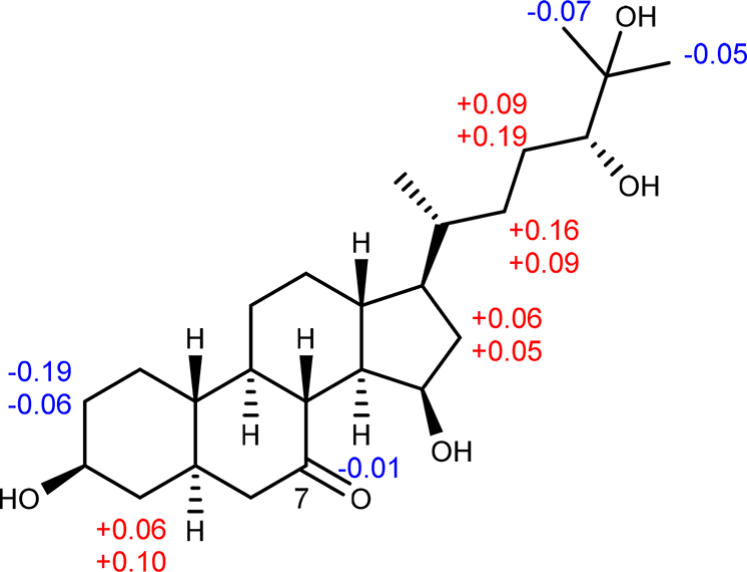
Δδ (δ_*S*_ – δ_*R*_) values (in ppm) derived from the chemical shifts of the (*S*)-MTPA and (*R*)-MTPA esters of **1**.

A crystal was obtained, and X-ray diffraction (XRD) data not only confirmed the proposed structure but also allowed the unambiguous assignment of the relative configuration of **1** (Figure 5). The relative configuration of **1** was consistent with previously reported naturally occurring steroids.^12,13^ To establish the absolute configuration at C-3, C-15, and C-24, the modified Mosher’s method was applied.^14^ According to a convenient Mosher ester procedure carried out in NMR tubes, the corresponding (*R*)- and (*S*)-MTPA esters (**1r** and **1s**) of **1** were prepared by treatment with (*S*)-MTPA-Cl and (*R*)-MTPA-Cl, respectively. After analysis of the ^1^H NMR and the ^1^H–^1^H COSY spectra, the chemical shift differences between the (*R*)- and (*S*)-MTPA esters (Δδ = δ**_1s_** – δ**_1r_**) were calculated, which suggested 3*S*, 15*R*, and 24*R* configurations (Figure 6). These data in conjunction with XRD analysis allowed the assignment of the absolute configuration of **1**. Thus, the structure of **1** was established as (3*S*,5*R*,15*R*,20*R*,24*R*)-3,15,24,25-tetrahydroxycholestan-7-one, to which we assigned the trivial name nannosterol A.

Nannosterol B (**2**) was purified as an amorphous white powder with the molecular formula of C_27_H_47_O_6_ as mentioned above. The NMR data of **2** were very similar to those of **1** except for the presence of an oxygenated methylene group at δ_H_ 3.63 (d, *J* = 12.2 Hz, H_2_-18a) and 3.85 (d, *J* = 12.2 Hz, H_2_-18b) in **2** instead of a methyl group in **1** (Table 1). This additional hydroxy group was deduced to be located at C-18 based on the HMBC correlation from H_2_-18a and H_2_-18b to C-12 (δ_C_ 37.9), C-13 (δ_C_ 47.9), C-14 (δ_C_ 55.2), and C-17 (δ_C_ 57.0) (Figure 3). Analysis of 1D and 2D NMR of **2** suggested the remaining substructure to be identical with that of **1**. Thus, compound **2**, to which we assigned the trivial name nannosterol B, was elucidated as a 18-hydroxy derivative of nannosterol A (**1**). From a biogenetic point of view, compound **2** should retain the same stereochemistry as that of nannosterol A (**1**), which is in congruence with the observed negative specific optical rotation for both compounds in MeOH ([α]^20^_D_).

### Bioactivity of Nannosterols

Nannosterols were not active against *Escherichia coli* DSM 1116^T^, *E. coli* JW0451-2 (*acrB*-efflux pump deletion mutant of *E. coli* BW25113), *Pseudomonas aeruginosa* PA14 (DSM 19882), *Bacillus subtilis* DSM10^T^, *Mycobacterium smegmatis* (DSM 43756), *Staphylococcus aureus* Newman, *Candida albicans* DSM 1665, or *Mucor hiemalis* DSM 2656 and showed no antiproliferative activity against human HepG2 hepatocellular carcinoma cells. Thus, the biological function of nannosterols remains to be discovered.

### *In Silico* Biosynthetic Investigation of Nannosterols

Although the production of modified sterols such as 5α-cholest-7-en-3β-ol (lathosterol) and cholest-8-en-3-ol has been previously described^15^ for myxobacteria, the discovery of **1** and **2** presents the first sterols exclusively produced by myxobacteria with chemical modifications unprecedented for prokaryotes. Therefore, an extended metabolome analysis was performed to investigate the occurrence of **1** and **2** across myxobacterial taxa, using a previously established collection of high-resolution LC-MS data sets from ca. 2600 myxobacterial strains.^11^ The findings of this survey confirmed that the nannosterols are exclusively produced within the myxobacterial suborder of Nannocystineae (Table S2, Figure S25) and present therefore an unnoticed bacterial sterol class with yet unknown biological function.

**Figure 7:**
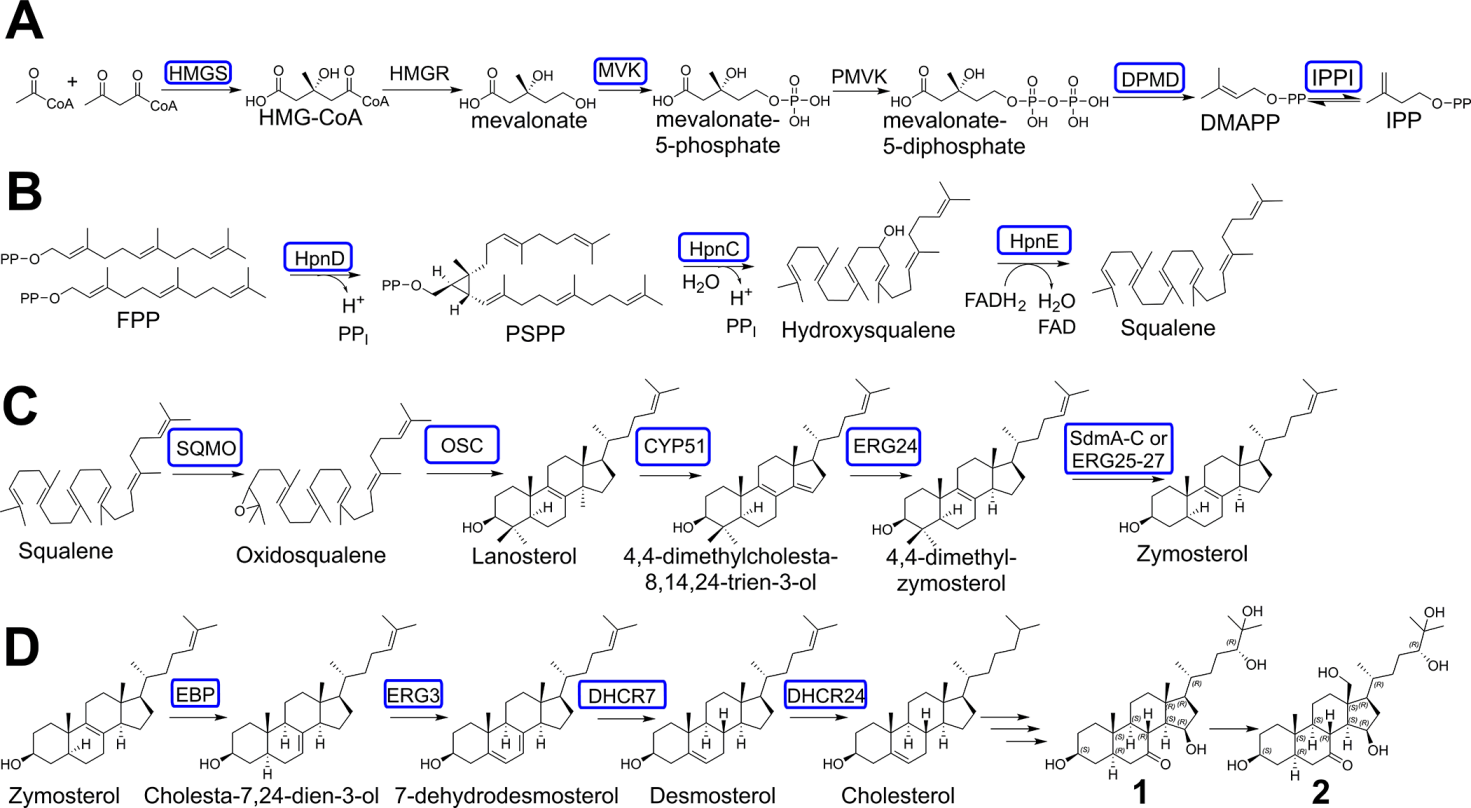
Proposed biosynthetic pathway leading to the formation of **1** and **2**. (A) DMAPP and IPP are probably formed via the mevalonate pathway. (B) The squalene formation is presumably catalyzed via a three-step pathway (HpnCDE) using the enzymes hydroxysqualene synthase (HpnC), presqualene diphosphate synthase (HpnD), and hydroxysqualene dehydroxylase. (C) The presence of different “stage 2” genes within the genome of Na p20, which encode numerous modification enzymes requiring molecular oxygen for catalysis,^10^ lead probably to the formation of 4,4-dimethylcholesta-8,14,24-trien-3-ol, zymosterol, and cholesta-7,24-dien-3-ol, which have been previously discovered from different myxobacterial strains.^32^ (D) Unidentified tailoring enzymes are likely decorating the sterol scaffold of cholesterol (or another closely related derivative) to yield **1** and **2**. Identified gene homologues of biosynthetic proteins within the genome of Na p20 are marked in blue boxes. HMGS: 3-hydroxy-3-methylglutaryl-coenzyme A synthase; HMG-CoA: 3-hydroxy-3-methylglutaryl- coenzyme A; MVK: mevalonate kinase; PMVK: phosphomevalonate kinase; DPMD: diphosphomevalonate decarboxylase/mevalonate-5-pyrophosphate decarboxylase; DMAPP: dimethylallyl diphosphate; IPPI: isopentenyl (pyro)/diphosphate isomerase; IPP: isopentenyl (pyro)/diphosphate; FPP: farnesyl diphosphate; HpnD: presqualene diphosphate synthase; PSPP: presqualene (pyro)/diphosphate; HpnC: hydroxysqualene synthase; HpnE: hydroxysqualene dehydroxylase; SQMO: squalene monooxygenase; OSC: oxidosqualene cyclase; CYP51: lanosterol 14-alpha demethylase/C-14 demethylase (ERG11 homologue); ERG24: delta(14)-sterol reductase; SdmA: sterol demethylase protein A/4beta-methylsterol monooxygenase; SdmB: sterol demethylase protein B/3beta-hydroxysteroid-4beta-carboxylate 3-dehydrogenase; SdmC: sterol demethylase protein C^20^; ERG25: C-4 methylsterol oxidase; ERG26: sterol-4-alpha-carboxylate 3-dehydrogenase; ERG27: C-3 keto sterol reductase; EBP: 3-beta-hydroxysteroid-delta(8),delta(7)-isomerase; ERG3: delta(7)-sterol 5(6)-desaturase/C-5 desaturase; DHCR7:7-dehydrocholesterol reductase; DHCR24: delta(24)-sterol reductase.

Based on the chemical structure of nannosterols together with previous biochemical and genetic studies investigating the biosynthesis of bacterial sterols,^10,16^ we postulate **1** and **2** to be synthesized by similar biosynthetic proteins. *In silico* analysis with the antibiotics and secondary metabolite analysis shell (antiSMASH)^17^ of the genome-sequenced alternative producer of **1** and **2***Nannocystis pusilla* Na p20 (DSM 53165) led to the identification of 10 putative terpene gene clusters (Table S3). According to retrobiosynthetic considerations, none of these identified gene clusters harbor all the biosynthetic genes required for the production of **1** and **2**. Therefore, we propose that the genes encoding the required biosynthetic proteins are distributed throughout the myxobacterial genome. This characteristic resembles the genetic organization of plant biosynthetic pathways in general^18,19^ and sterol pathways within the bacterial domain.^16,20^ Terpenes are in general synthesized through activated monomers of isoprene, namely, isopentenyl (pyro)/diphosphate (IPP) and dimethylallyl diphosphate (DMAPP), which are generated either by the mevalonate (or 3-hydroxy-3-methylglutaryl-coenzyme A (HMG-CoA) reductase pathway^21^ or the 2-*C*-methyl-d-erythritol 4-phosphate/1-deoxy-d-xylulose-5-phosphate (MEP/DOXP)/nonmevalonate pathway.^22^ In the genome sequence of *N. pusilla* Na p20 homologues of HMG-CoA synthase, mevalonate kinase (MVK), mevalonate-5-pyrophosphate decarboxylase and IPP isomerase were found. Although no obvious gene homologue of a phosphomevalonate kinase or HMG-CoA reductase was identified—in contrast to the previously investigated myxobacterial strains^23^*Myxococcus xanthus* DK1622^24^ and *Stigmatella aurantiaca* DW4/3-1^25^—these findings strongly support the potential of *N. pusilla* Na p20 to produce DMAPP and IPP via the mevalonate pathway (Figure 7A).

Consequently, the triterpene scaffold required for the biosynthesis of **1** and **2** can be generated by an oligoprenyl synthetase to elongate via a head-to-tail condensation of DMAPP with two IPP monomers to form farnesyl diphosphate (FPP). The prenyl chain elongation can proceed by either a single-step pathway catalyzed by a squalene synthase (SQS) or a three-step pathway (HpnCDE) using the enzymes hydroxysqualene synthase (HpnC), presqualene diphosphate synthase (HpnD), and hydroxysqualene dehydroxylase (HpnE).^26,27^ Since gene homologues encoding oligoprenyl synthetase and gene homologues of *hpnC*, *hpnD*, and *hpnE* are present in the genome sequence of *N. pusilla* Na p20, the formation of squalene seems to be a reasonable assumption (Figure 7B). A previous study investigating bacterial triterpene biosynthesis divides steroid biosynthesis into two stages; stage 1 (also termed steroid precursor biosynthesis) leads to the oxygen-independent formation of squalene, whereas stage 2 includes steroid cyclization and further modification reactions requiring molecular oxygen.^10^ Stage 2 initiation typically involves a gene encoding a squalene monooxygenase [SQMO, or an alternative SQMO termed AltSQMO] and an oxidosqualene cyclase (OSC) to form the tetracyclic triterpenoid scaffolds represented by lanosterol and cycloartenol. Previous site-directed mutagenesis studies have identified three amino acid changes that seem to be influential in the production profile of OSC.^28−30^ Further gene sequence analysis revealed that simply one of these residues was conserved and suggested that a valine (V) or isoleucine (I) at residue 453 indicated lanosterol or cycloartenol production.^31^ A gene homologue of SQMO and OSC was identified in DSM 53165; since the identified OSC has V453, we propose that it catalyzes the production of lanosterol (Figures S26, S27). Since gene homologues for CYP51 (C-14 demethylase), ERG24 (delta(14)-sterol reductase), SdmA–C^20^ (sterol demethylase protein A–C), and ERG25–27 (ERG25: C-4 methylsterol oxidase; ERG26: sterol-4-alpha-carboxylate 3-dehydrogenase; ERG27: C-3 keto sterol reductase) were identified in the genome sequence of *N. pusilla* Na p20, the biosynthesis might include the formation of the intermediates 4,4-dimethylcholesta-8,14,24-trienol, 4,4-dimethylzymosterol, and zymosterol (5α-cholesta-8,24-dien-3β-ol), which have been previously discovered from different myxobacterial strains from the suborder Nannocystineae (zymosterol and 4,4-dimethylzymosterol^15^) (Figure 7C).

The presence of gene homologues encoding EBP (3-beta-hydroxysteroid-delta(8),delta(7)-isomerase), ERG3 (C-5 desaturase), DHCR7 (7-dehydrocholesterol reductase), and DHCR24 (delta(24)-sterol reductase) within the genome of *N. pusilla* Na p20 indicates that cholesterol might be the precursor of **1** and **2** (Figure 7D). It is also possible that a different sterol intermediate (especially a precursor of cholesterol) such as zymostenol might be produced by *N. pusilla* Na p20—as described previously for *Nannocystis excedens*(32)—and used as precursor for **1** and **2**. Nevertheless, we hypothesize that **1** and **2** are biosynthesized by a hydroxylation cascade catalyzed by different cytochrome P450 enzymes (CYP450s), starting from a closely related derivative of the major sterol intermediate zymosterol (Figure 7D). The hydroxylation pattern of the nannosterol formation is unprecedented for bacterial sterol biosynthesis, which includes hydroxylations at position C-15, at C-24–C-25 to form a terminal vicinal diol side chain, and at the angular methyl group at C-18, which presumably happens at the end of the nannosterol biosynthesis. While hydroxylation at position C-15 has been described previously from bacterial CYP450s^33^ in particular catalyzed by the subfamilies CYP106A1, CYP106B1,^34^ and CYP109B1, hydroxylation at the angular methyl group at C-18 is unprecedented for bacterial steroids. In humans, the enzyme CYP11B2^35^—known as aldosterone synthase—catalyzes sequential hydroxylations of the angular methyl group at C-18 of different steroids and has therefore a central role during the biosynthesis of mineralocorticoid aldosterone and other steroids.^36^

Another rare chemical feature of these natural products is the C-7 position of the ketone group that has only been reported in natural products isolated from sponge-associated *Psychrobacter* sp.^37^ and *Hasllibacter halocynthiae*(38) and from the marine-derived actinomycete *Streptomyces seoulensis*.^39^ The formation of the ketone group at position C-7 might also be catalyzed by CYP450 to install first a hydroxy intermediate. Afterward, this hydroxy intermediate might be transformed to a ketone by the action of a hydroxysteroid dehydrogenase, which has been described for the positions C-3, C-11, C-17, and C-20^40^ but to the best of our knowledge not for position C-7. Since genetic inactivation in the producing myxobacterial strains has to date not been successful, mainly because *Nannocystis* sp. MNa10993 or *N. pusilla* strain DSM 53165 does not grow in suspension, it was not possible to unambiguously identify the genes encoding the biosynthetic tailoring enzymes to form **1** and **2**. In fact, the myxobacterial suborder Nannocystineae to date remains resistant to genetic manipulation in contrast to members of suborder Cystobacterineae such as the myxobacterial model host *Myxococcus xanthus* DK1622.^41^ Nevertheless, the uniqueness of this pathway may trigger future investigations using *in vitro* reconstituted enzymatic machineries or transplantation of the potential pathway genes into a heterologous host.

## Conclusion and Outlook

In summary, nannosterols constitute the first sterols exclusively produced by myxobacteria with chemical modifications up-to-date unprecedented for prokaryotes. The discovery that the production of this compound class is conserved within the suborder of Nannocystineae further emphasizes evolutionary differences between prokaryotic and eukaryotic sterol biogenesis. Thus, the discovery of nannosterols sets the stage for further in-depth genetic and biochemical analysis to shed light on the biosynthesis of bacterial sterols.

## Experimental Section

### General Experimental Procedures

^1^H NMR, ^13^C NMR, and 2D spectra were recorded at 700 MHz (^1^H)/175 MHz (^13^C), conducting with an Ascend 700 spectrometer using a cryogenically cooled triple resonance probe (Bruker Biospin, Rheinstetten, Germany). Samples were dissolved in MeOD_4_ and DMSO-*d*_6_. Chemical shifts are reported in ppm relative to tetramethylsilane; the solvent was used as the internal standard (SI 2.1 and 2.2, Figures S6–S23). Chiroptical rotation of **1** and **2** was measured in MeOH using the polarimeter model 341 (PerkinElmer Inc., Waltham, MA, USA) in a 50 mm × 2 mm cell at 25 °C ([α]^25^_D_).

### Isolation and Cultivation of MNa10993

The strain MNa10993 was recognized as a myxobacterium on standard mineral salt medium by agar-degrading swarm colony, bacterial predation, and formation of small solitary fruiting bodies. After a series of swarm purification steps, the strain was finally isolated. Phylogenetic analysis based on the 16S rRNA gene sequence revealed the strain’s position within the *Nannocystis* clade, which shows 99.32–99.28% similarity to *Nannocystis exedens* DSM 71^T^ (GenBank accession: NR_040928) and *Nannocystis pusilla* Na p29^T^ (GenBank accession: NR_104789), respectively (Figure S24). The myxobacterial strain MNa10993 was routinely cultivated at 30 °C in 24 L of CYH medium [%, (w/v), 0.1 g soya meal starch, (Sigma-Aldrich), 0.15 g Casitone (BD), 0.4 g soluble starch (Roth), 0.15 g yeast extract (BD), 0.1 g CaCl_2_, 0.005 g MgSO_4_·7H_2_O, 25 mM HEPES, 8 mg/L Fe-EDTA, pH adjusted to 7.3 with 10 N KOH before autoclaving] containing 5% (v/v) cell inoculum and 2% (v/v) sterile Amberlite resin XAD-16 (Sigma-Aldrich Chemie GmbH, Taufkirchen, Germany) for 10 d at 160 rpm. At the end of the fermentation, resin and cells were harvested together by centrifugation at 8000 rpm, for 30 min at 4 °C.

### Phylogenetic Analysis of MNa10993

For the isolation of genomic DNA from the myxobacterial strain MNa10993, the cells were obtained from an actively growing CYH culture. The genomic DNA was subsequently extracted following the standard method for Gram-negative bacteria using the Puregene Core kit A from Qiagen. PCR-based amplification of the 16S rRNA gene was performed using the universal primers f27 (5′-AGAGTTTGATCCTGGCTCAG-3′)^42^ and r1525 (5′-AAGGAGGTGATCCAGCCGCA-3′).^43^ PCRs were carried out in a Mastercycler Pro (Eppendorf) using Phusion High-Fidelity according to the manufacturer’s protocol. The amplified PCR products were purified by agarose gel electrophoresis (0.8% (w/v) agarose, at 70 V, for 45 min) and isolated using a Macherey Nagel Nucleo Spin kit. Primers used for sequencing were r336 (5′-ACTGCTGCSYCCCGTAGGAGTCT-3′),^44^ r460 5′-AGCAGCCGCGGTAATACGG-3′),^45^ r518 (5′-CGT ATT ACC GCG GCT GCT GG-3′), f1100 (5′-YAACGAGCGCAACCC-3′), and r1100 (5′-GGGTTGCGCTCGTTG-3′).^43,46^ The 16S rRNA gene sequence (SI 4.1) was used to build a phylogenetic tree with the embedded Geneious software tool (Figure S24).

### Analysis of Secondary Metabolism of Broth Extracts

The broth extracts were analyzed by high-performance liquid chromatography–high-resolution electrospray ionization-diode array-detector–mass spectrometry (HPLC-HRESI-DAD-MS) on a maXis 4G mass spectrometer (Bruker Daltonics, Billerica, MA, USA) coupled with a Dionex UltiMate 3000 Rapid Separation (RS)LC system (Thermo Fisher Scientific, Waltham, MA, USA) using a BEH C_18_ column (100 × 2.1 mm, 1.7 μm) (Waters, Eschborn, Germany) with a gradient of 5–95% acetonitrile (ACN) + 0.1% formic acid (FA) in H_2_O + 0.1% FA at 0.6 mL/min and 45 °C over 18 min with ultraviolet (UV) detection by a diode array detector (DAD) at 200–600 nm. Mass spectra were acquired from 150 to 2000 *m*/*z* at 2 Hz. Detection was performed in the positive MS mode. The plugin for Chromeleon Xpress (Thermo Fisher Scientific, version 6.8) was used for operation of the Dionex UltiMate 3000 RSLC system. HyStar (Bruker Daltonics, version 3.2) was used to operate on the maXis 4G mass spectrometer system. HPLC-MS mass spectra were analyzed with DataAnalysis (Bruker Daltonics, version 4.2). In order to conduct statistical metabolome analysis to identify alternative producers of **1** and **2**, both the myxobacterial strain and medium blanks were cultivated and extracted in triplicates as described above. Each crude extract was measured as technical duplicates yielding a total number of six replicates for the bacterial and medium blank extracts. The T-ReX-3D molecular feature finder of MetaboScape 6.0.2 (Bruker Daltonics) was used to obtain molecular features. Detection parameters were set to intensity threshold 5 × 10^3^ and a minimum peak length of five spectra. Identification of bacterial features was performed with the built-in *t* test routine and filtered to appearance in all six bacterial extracts and in none of medium blank extracts. The in-house standard extract database embedded in the software bundle Mxbase Explorer 3.2.27 was used for the search of alternative producers of **1** and **2**.^11^ The chosen parameters to evaluate those MS data sets considering the exact mass (exact mass deviation below 5 ppm), isotope pattern, and retention time matching (retention time deviation below 0.3 min) were adapted from previous studies investigating the presence of different myxochelin congeners.^47,48^

### Isolation of **1** and **2** by Semipreparative HPLC

The extraction, isolation, and purification of **1** and **2** from the myxobacterial broth was initiated by liquid–liquid extraction to concentrate the nannosterols in the chloroform (CHCl_3_) and ethyl acetate (EA) phase. Subsequent fractionation of these extracts by flash chromatography and further purifications of these resulted in different fractions containing **1** and **2**. Further processing via semipreparative HPLC yielded pure compound **1**, **2**. Similar compound isolation procedures from myxobacterial broth have been described previously.^49,50^ The cell pellet and XAD-16 resin (obtained by centrifugation) were extracted by acetone elution and subsequently evaporated under vacuum (6.9 g). The extract was then partitioned between MeOH and *n*-hexane solvents. The MeOH layer was dried under vacuum to yield 4.5 g of extract. This extract was partitioned in water using chloroform (CHCl_3_) and EA to yield 282 mg and 480 mg, respectively, after *invacuo* solvent evaporation. The EA extract (489 mg) was then subjected to flash chromatography on an Isolera One (Biotage, Uppsala, Sweden) with a SNAP 100 g column packed with reverse phase silica gel (C_18_) (90 Å, 200–400 mesh, 40–63 μm), using H_2_O + 0.1% FA as solvent A, MeOH + 0.1% FA as solvent B, and acetone + 0.1% FA as solvent C. The flow rate was 50 mL/min, UV/vis absorption was set at 270 and 335 nm. Collected fractions (45 mL) were monitored on a Dionex UltiMate 3000 RSLC system (Thermo Fisher Scientific) coupled to an amaZon ion trap MS (Bruker Daltonics) coupled to an amaZon ion trap MS (Bruker Daltonics). The elution gradient consisted of an initial isocratic mixture of 95:5 H_2_O–MeOH for five column volumes (CVs), then raised to 5:95 H_2_O–MeOH for 20 CV. This was followed by another isocratic solvent system to 5:95 H_2_O–MeOH for eight CVs. A final gradient of 5:95 MeOH–acetone was reached after five CVs. Using high-resolution mass spectrometry, two fractions containing compounds of similar masses and related retention times were pooled together and dried under vacuum; fractions 54–56 (126 mg) contained A, and fractions 57–59 (200 mg) contained B. These fractions were separately purified on an UltiMate 3000 semipreparative system coupled to a Thermo Scientific Dionex UltiMate 3000 series automated fraction collector (Bruker Daltonics) using a C_18_ Phenomenex Luna (100 Å, 5 μm, 10 × 250 mm) LC column (Phenomenex, Torrance, CA, USA) and eluted with H_2_O + 0.1% FA and ACN + 0.1% FA. The fractions were monitored by mass spectrometry and by using the UV/vis detector set at 220, 280, 320, and 400 nm. The gradient program was set to an initial isocratic gradient of 60:40 (H_2_O–ACN) for 5 min followed by a gradient ramp to 30:70 H_2_O–ACN in 5 min. The gradient was then maintained at 30:70 H_2_O–ACN for 18 min before being raised again to 5:95 H_2_O–ACN in 5 min and held for 2 min before lowering the gradient back to 95:5 H_2_O–ACN in 1 min. The column was re-equilibrated for 5 min using 95:5 H_2_O–ACN. The compounds were detected using mass spectrometry on the Agilent 1100 series (Agilent Technologies, Santa Clara, CA, USA) coupled to the HCT 3D ion trap (Bruker Daltonics) or with a UV detector on the Dionex UltiMate 3000 RSLC system by UV absorption at 220, 260, 320, and 400 nm. The HPLC fractions were dried under N_2_. Compound **1** (2.0 mg) from fraction 54–56 eluted at a retention time of 8.53 min, and compound **2** (3.5 mg) from fraction 57–59 eluted at retention time 6.92 min.

### Nannosterol A (**1**)

Amorphous white powder; HRESIMS *m*/*z* 451.3421 [M + H]^+^ (calcd for C_27_ H_47_O_5_, 451.34180), retention time 8.5 min, [α]^20^_D_ = −10.4 (*c* 0.1, MeOH); ^1^H and ^13^C NMR data, Table 1.

### Nannosterol B (**2**)

Amorphous white powder; HRESIMS *m*/*z* 467.3357 [M + H]^+^ (calcd for C_27_ H_47_O_6_, 467.33672), retention time 6.5 min, [α]^20^_D_ = −4.0 (*c* 0.1, MeOH); ^1^H and ^13^C NMR data, Table 1.

### Preparation of (*R*)- and (*S*)-MTPA Esters

According to a previously described protocol, the (*S*)- and (*R*)-MTPA ester derivatives of **1** were prepared.^14^ In brief, compound **1** (0.5 mg, 0.001 mmol, 98%) and dry pyridine (10 μL, 125 μmol, 39 equiv, 99%) were transferred separately in a 2 mL glass vial followed by the addition of anhydrous CDCl_3_ (100 μL). Afterward, (*R*)-MTPA-Cl (10 μL, 5.2 μmol, 16 equiv) was added to the vial, and the resulting mixture was stirred at room temperature for 2 h. After completion of the reaction, the reaction mixture was diluted with dry CDCl_3_ (0.6 mL). The entire CDCl_3_ solution was then transferred to a standard NMR tube, and the ^1^H NMR spectrum of the resulting (*S*)-MTPA ester was recorded. The assignment of the chemical shifts was made on the basis of the ^1^H–^1^H COSY spectra. Following a similar procedure, the (*R*)-MTPA ester was obtained by using (*S*)-MTPA-Cl.

### Crystallization Parameters and X-ray Crystallographic Data of **1**

From a solution of methanol and cyclohexane (1:10), **1** was obtained as colorless needles. The data set was collected using a Bruker D8 Venture diffractometer with a microfocus sealed tube and a Photon II detector. Monochromated Cu Kα radiation (λ = 1.541 78 Å) was used. Data were collected at 133(2) K and corrected for absorption effects using the multiscan method. The structure was solved by direct methods using SHELXT^51^ and was refined by full matrix least-squares calculations on *F*^2^ (SHELXL2018)^52^ in the graphical user interface Shelxle.^53^ All relevant data regarding the crystal structure of **1** can be found in the SI (SI 3, Table S1). Crystallographic data for the structure have been deposited with the Cambridge Crystallographic Data Centre, CCDC, 12 Union Road, Cambridge CB21EZ, UK. Copies of the data can be obtained free of charge on quoting the repository number 2223627 www.ccdc.cam.ac.uk/data_request/ci.

### Bioactivity Profiling of **1** and **2**

For evaluation of antibacterial and antifungal activities of compounds **1** and **2**, *E. coli* DSM 1116^T^, *E. coli* JW0451-2 (*acrB*-efflux pump deletion mutant of *E. coli* BW25113), *P. aeruginosa* PA14, *B. subtilis* DSM10^T^, *M. smegmatis* DSM 43756, *S. aureus* Newman, *C. albicans* DSM 1665, and *M. hiemalis* DSM 2656 were assayed using the microbroth dilution assay as described previously.^54^ These strains present a representative selection of bacterial and fungal microorganisms to evaluate biological activity of natural products. Cytotoxic activity of compounds was determined using HepG2 hepatocellular carcinoma cells seeded at 6 × 10^3^ cells per well of 96-well plates in 180 μL of complete medium and treated with test compounds in serial dilution after 2 h of equilibration. After 5 days of incubation, 20 μL of 5 mg/mL MTT (thiazolyl blue tetrazolium bromide) in phosphate-buffered saline (PBS) was added per well, and it was further incubated for 2 h at 37 °C. The medium was discarded, and cells were washed with 100 μL of PBS before adding 100 μL of isopropanol/10 N HCl (250:1) in order to dissolve formazan granules. The absorbance at 570 nm was measured using the microplate reader Infinite M200Pro (Tecan Group Ltd., Männedorf, Switzerland), and cell viability was expressed as a percentage relative to the respective MeOH control. IC_50_ values were determined by sigmoidal curve fitting.

### Applied Software, DNA Sequence Analysis, and Bioinformatics Methods

The available genome sequence of *N. pusilla* Na p20 (DSM 53165) was screened for secondary metabolite BGCs using the antiSMASH 6.0^17^ online tool and the software Geneious Prime (Biomatters Ltd., Auckland, New Zealand, 2020.0.5).^55^ The nucleotide or amino acid sequence of interest was aligned with the basic local alignment search tool (BLAST) against our in-house genome database or the publicly available nucleotide database, in order to find homologous genes or proteins. The functional prediction of ORFs was performed by using either protein blast and/or blastx programs and Pfam.^56^ To obtain further information concerning the catalytic function of the identified biosynthetic proteins, the amino acid sequences were evaluated by the *in silico* protein homology analogy recognition engine 2 (Phyre2).^57^ Raw data from the alignments for *in silico* evaluation of the nannosterol biosynthetic proteins were stored on the in-house server. Sequence alignments were performed with the embedded Geneious alignment software with the following setups: Pairwise alignments (alignment type: global alignment with free end gaps; cost matrix: Blosum62; gap open penalty: 12; gap extension penalty: 3). Multiple alignments (alignment type: global alignment with free end gaps; cost matrix: Blosum45; gap open penalty: 12; gap extension penalty: 3; refinement iterations: 2). Further information concerning gene sequences can be found in the Supporting Information.

## Supporting Information Available

Spectra of **1** and **2**; crystallographic data of **1** (PDF)
X-ray crystallographic data for **1** (CIF)

## Supplementary Material

np2c01143_si_003.pdf

np2c01143_si_004.cif
