# Supplementary material for: Structure Elucidation and Biosynthesis of Nannosterols A and B, Myxobacterial Sterols from Nannocystis sp. MNa10993
Source: J Nat Prod. 2023 Apr 3;86(4):915–23. doi: 10.1021/acs.jnatprod.2c01143 (PMC10152446; doi:10.1021/acs.jnatprod.2c01143)
Supplement: Supplementary file 1 — np2c01143_si_003.pdf [file np2c01143_si_003.pdf]

Supplementary Information

Structure Elucidation and Biosynthesis of Nannosterols A and B; Myxobacterial Sterols from Nannocystis sp. MNa10993

Sergi H. Akone[†,‡,§,⊥,∥,ǂ], *Joachim J. Hug*[†,‡,§,⊥,ǂ], *Amninder Kaur*[†,‡,§,⊥], *Ronald Garcia*[†,‡,§,⊥], *and Rolf Müller*[†,‡,§,⊥,*]

[†]*Helmholtz-Institute for Pharmaceutical Research Saarland (HIPS), Helmholtz Centre for Infection Research (HZI), Department of Microbial Natural Products, Saarland University, Campus E8 1, 66123 Saarbrücken, Germany*

[‡]*Department of Pharmacy, Saarland University, Campus E8 1, 66123 Saarbrücken, Germany;*

[§]*German Center for Infection Research (DZIF), Partner Site Hannover-Braunschweig, 38124 Braunschweig, Germany*

[⊥]*Helmholtz International Labs, Department of Microbial Natural Products, Saarland University, Campus E8 1, 66123 Saarbrücken, Germany*

[∥]*Department of Chemistry, Faculty of Science, University of Douala, Douala, P.O. Box 24157, Cameroon.*

[ǂ]These authors contributed equally to this work.

* Author to whom correspondence should be addressed: rolf.mueller@helmholtz-hips.de

Table of contents

1. MS spectra.. 3

1.1 Partial ESI-MS spectra.. 3

1.2 MS^2 fragmentation spectra 5

2. NMR spectroscopic data for **1** and **2** 6

2.1 NMR spectra of Nannosterol A (**1**) 6

2.2 NMR spectra of Nannosterol B (**2**)...14

3. Crystallographic data of **1** 17

4. Genetic and biosynthetic investigations..19

1. MS spectra

1.1 Partial ESI-MS spectra

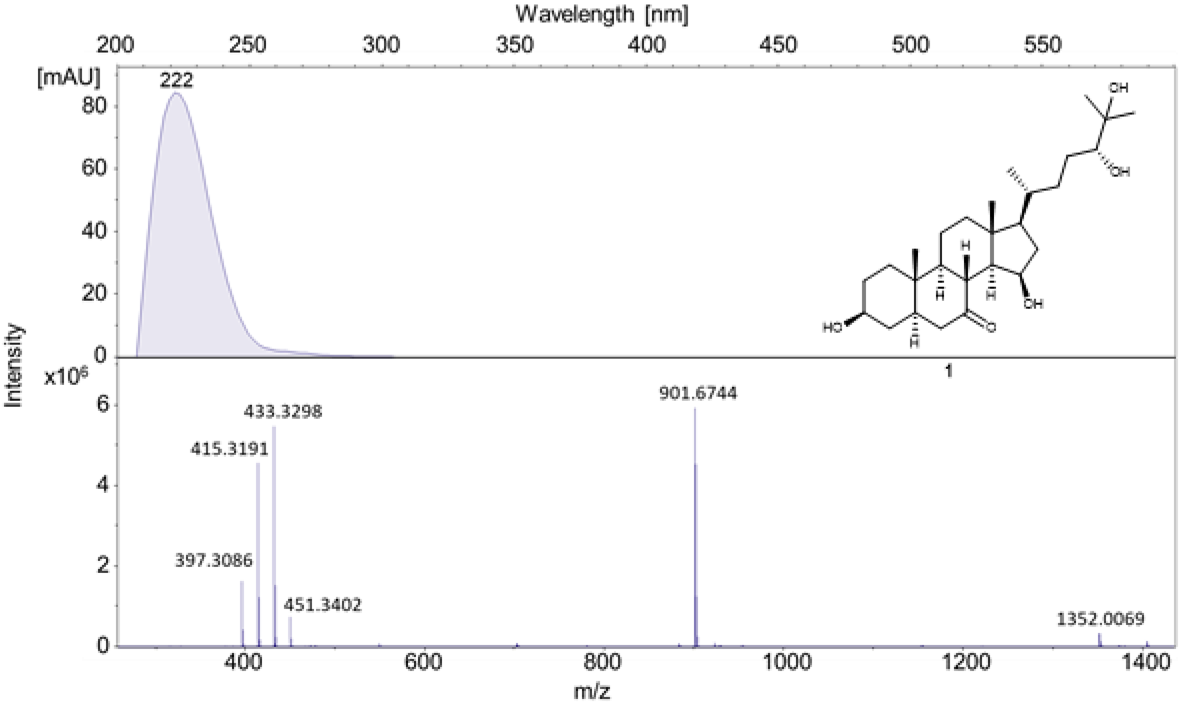

Figure S1. UV/VIS and partial ESI+MS spectra of purified **1** (451.3402 $[M+H]^+$, 433.3313 $[M-H_2O+H]^+$, 415.3191 $[M-2H_2O+H]^+$, 397.3086 $[M-3H_2O+H]^+$, 901.3313 $[2M+H]^+$, 1352.0069 $[3M+H]^+$.

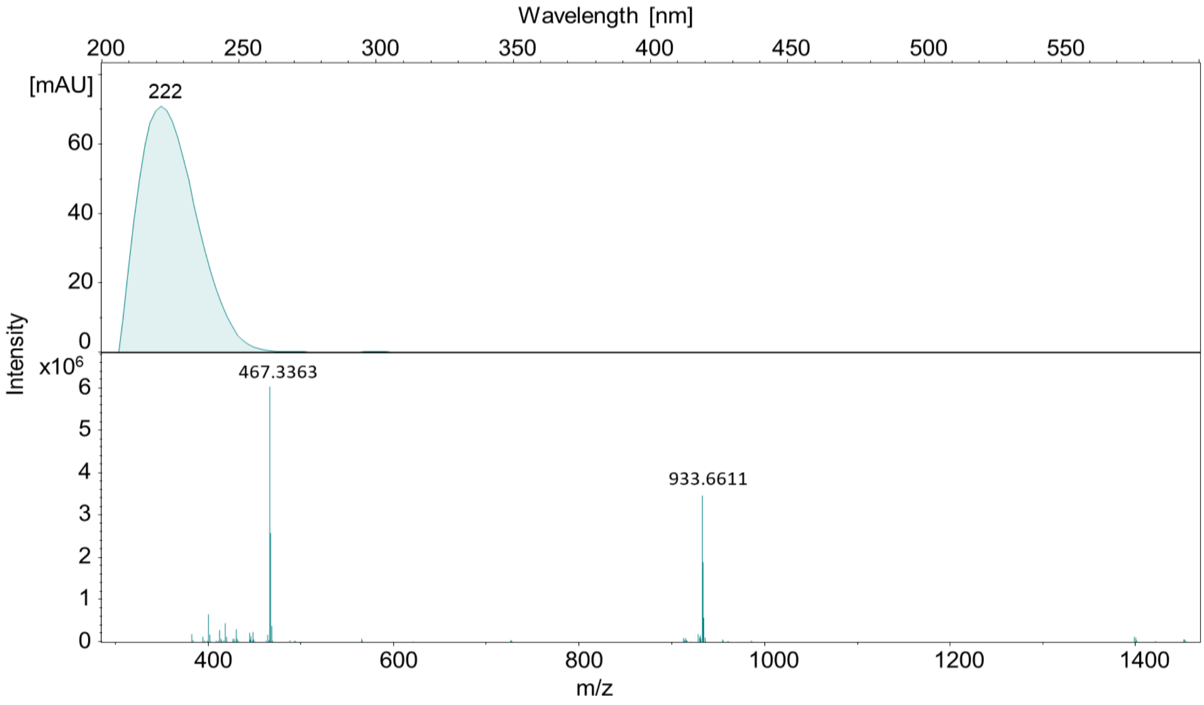

Figure S2. UV/VIS and partial ESI+MS spectra of purified **2** (467.3363 [M+H], 933.6611 $[2M+H]^+$.

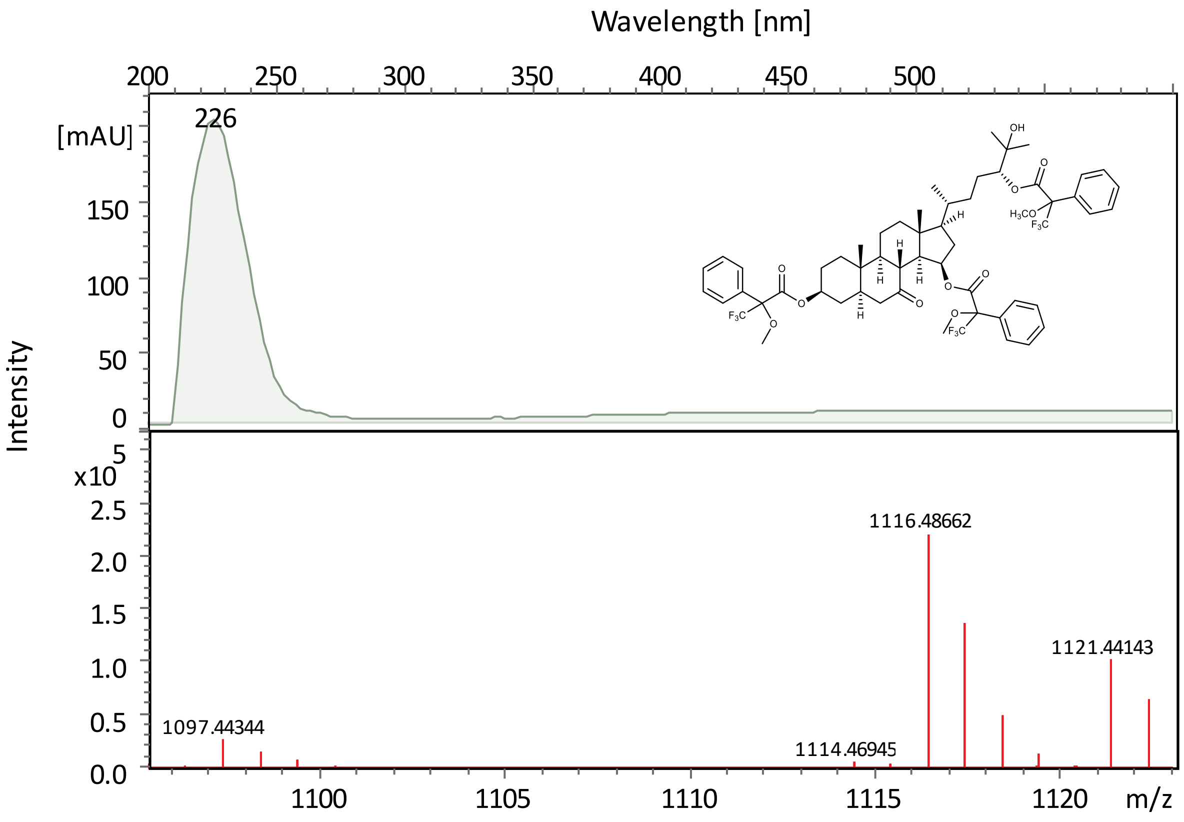

Figure S3. UV/VIS and partial ESI+MS spectra of (S)-MPTA ester of **1** (1099.4516 [M+H], 1116.4866 [(M+H_2O)+H]$^+$, 1121.4414 [M+Na]$^+$.

1.2 MS[2] fragmentation spectra

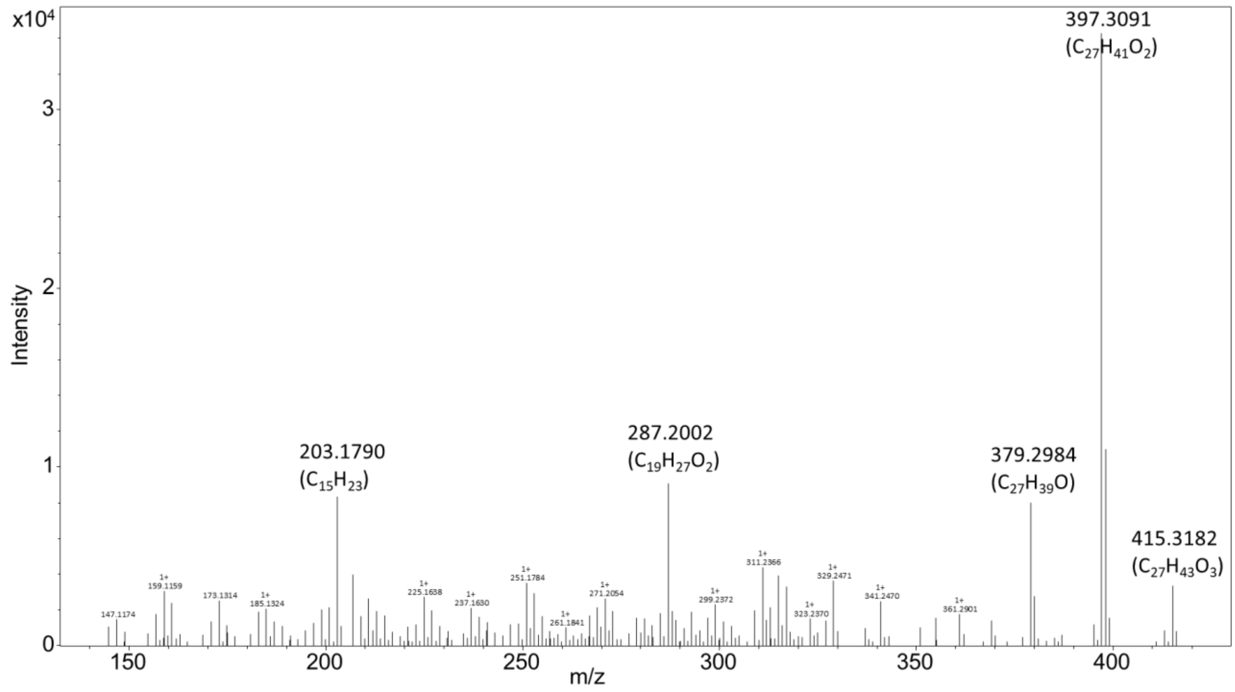

Figure S4. Fragmentation pattern in ESI+MS^2 experiment of Nannosterol A (**1**) (Collision energy: 28.0 –42.0 eV) and calculated sum formula of MS^2 fragments.

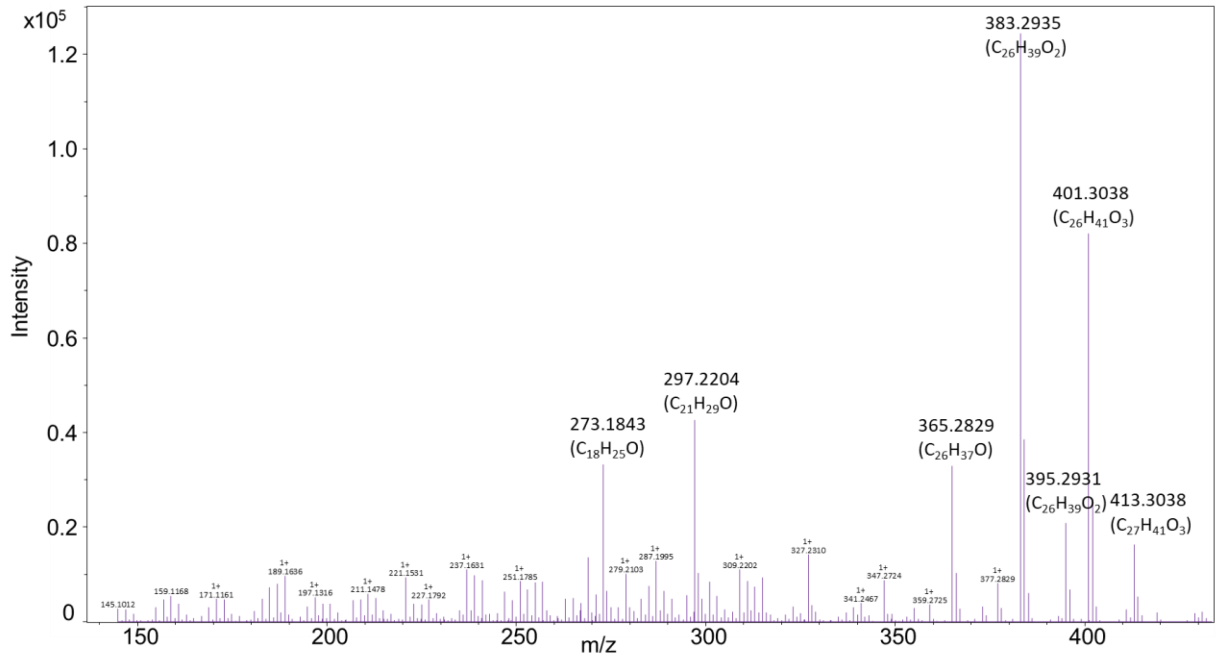

Figure S5. Fragmentation pattern in ESI+MS^2 experiment of nannosterol B (**2**) (Collision energy: 28.0 – 42.0 eV) and calculated sum formula of MS^2 fragments.

2. NMR spectroscopic data for **1** and **2**

2.1 NMR spectra of Nannosterol A (**1**)

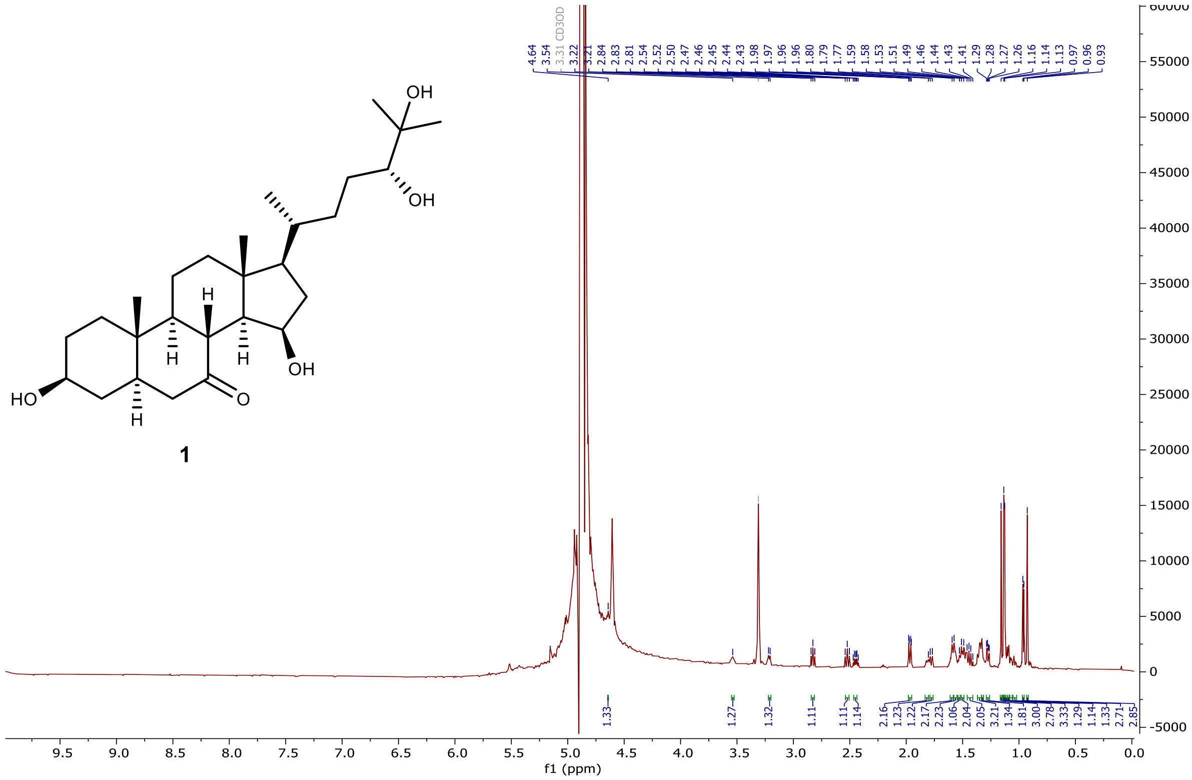

Figure S6. ^{1}H-NMR spectrum of nannosterol A (**1**) in CD_3OD (700 MHz).

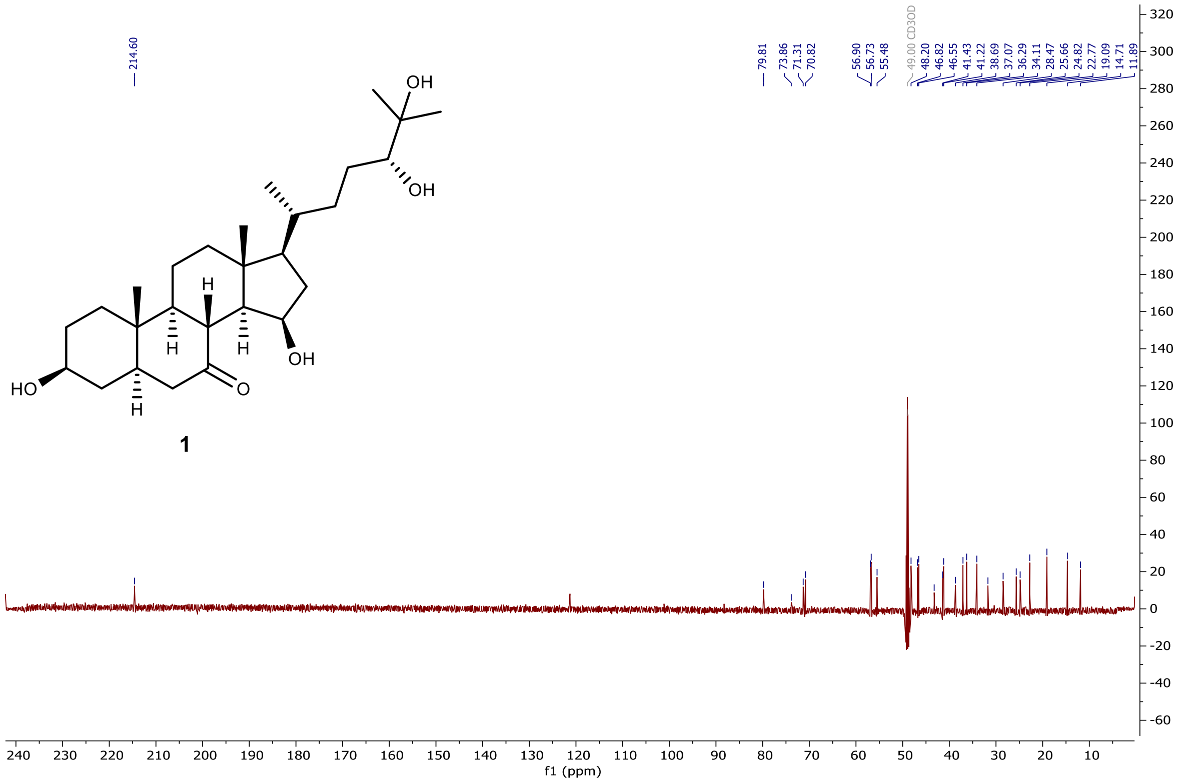

Figure S7. ^{13}C-NMR spectrum of nannosterol A (**1**) in CD_3OD (175 MHz).

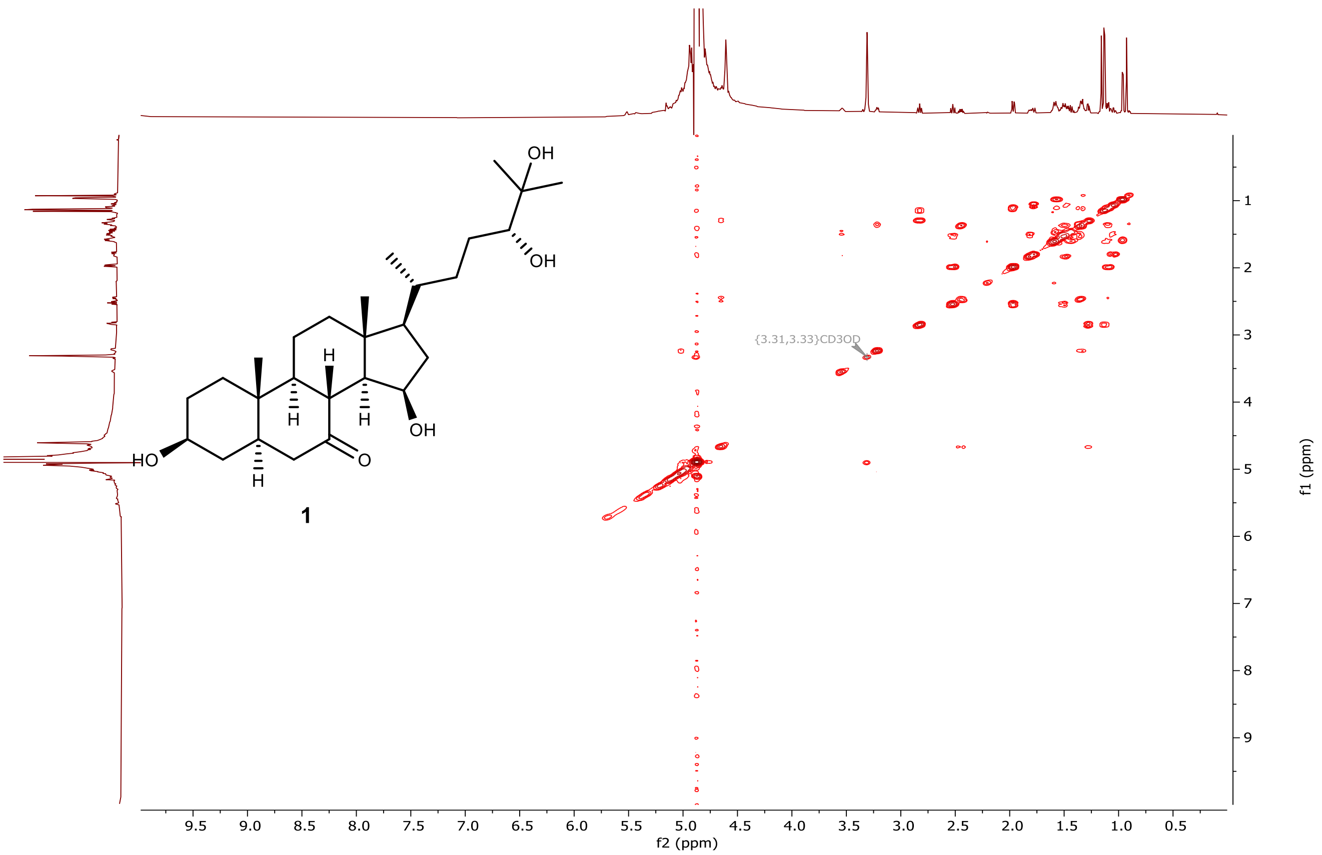

Figure S8. ^1H-^1H COSY spectrum of nannosterol A (**1**) in CD_3OD (700 MHz).

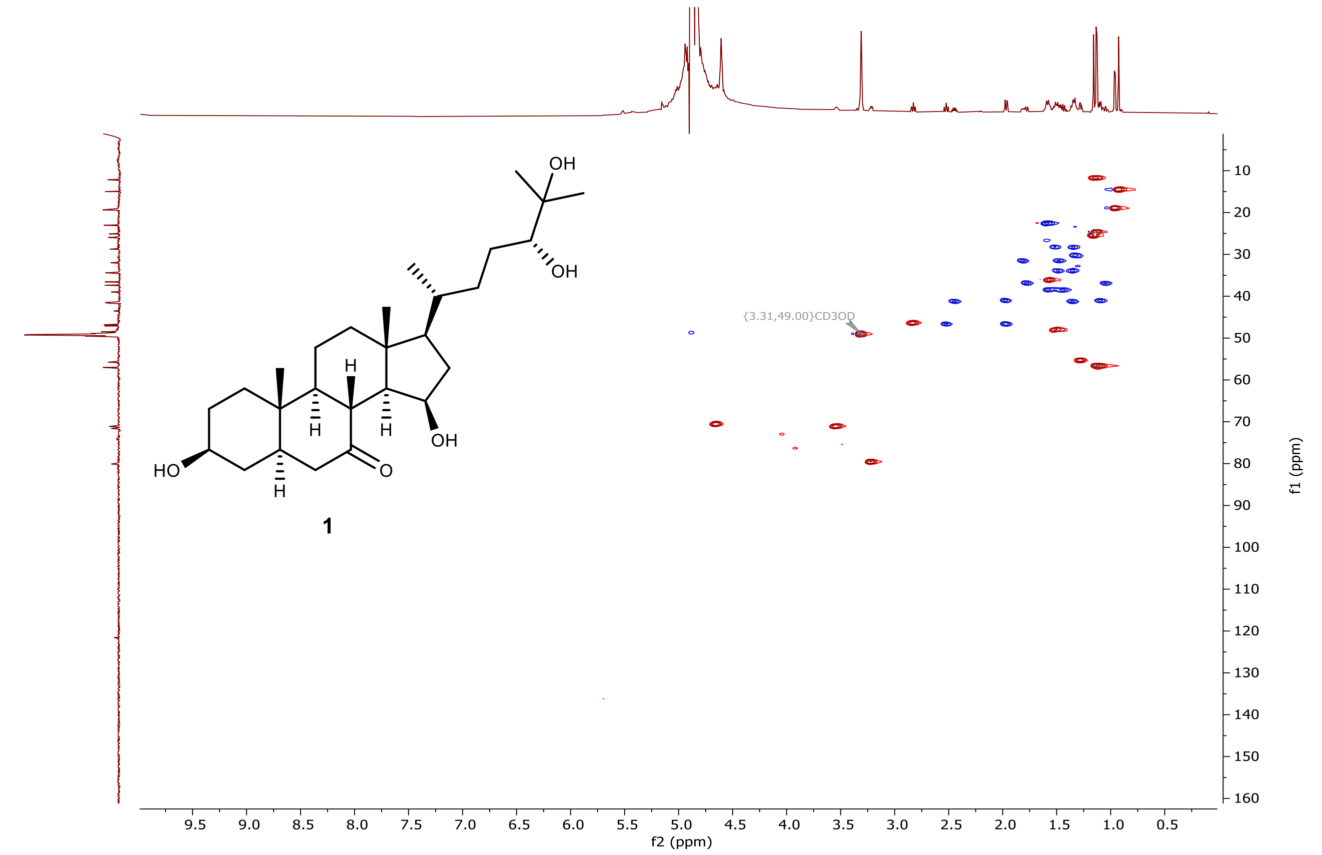

Figure S9. HSQC spectrum of nannosterol A (**1**) in CD_3OD (700 MHz and 175 MHz).

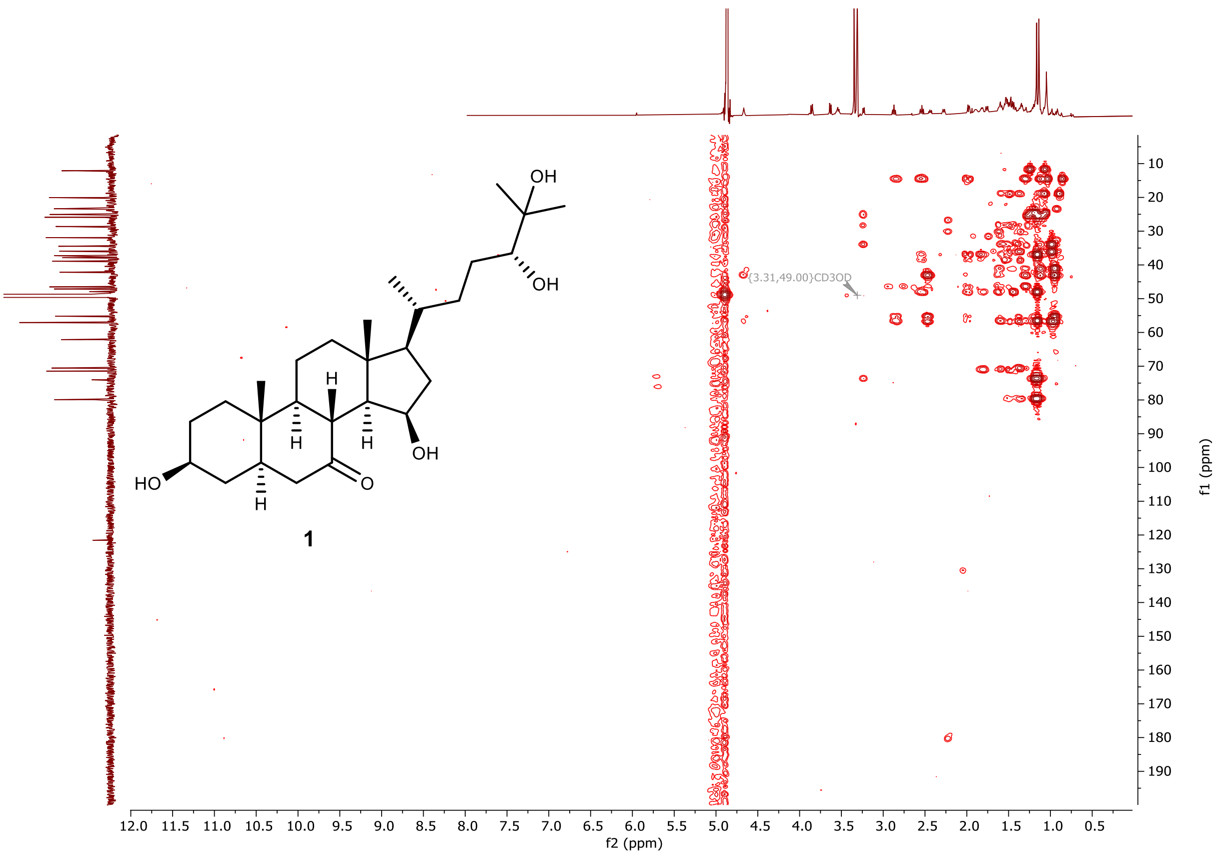

Figure S10. HMBC spectrum of nannosterol A (**1**) in CD_3OD (700 MHz and 175 MHz).

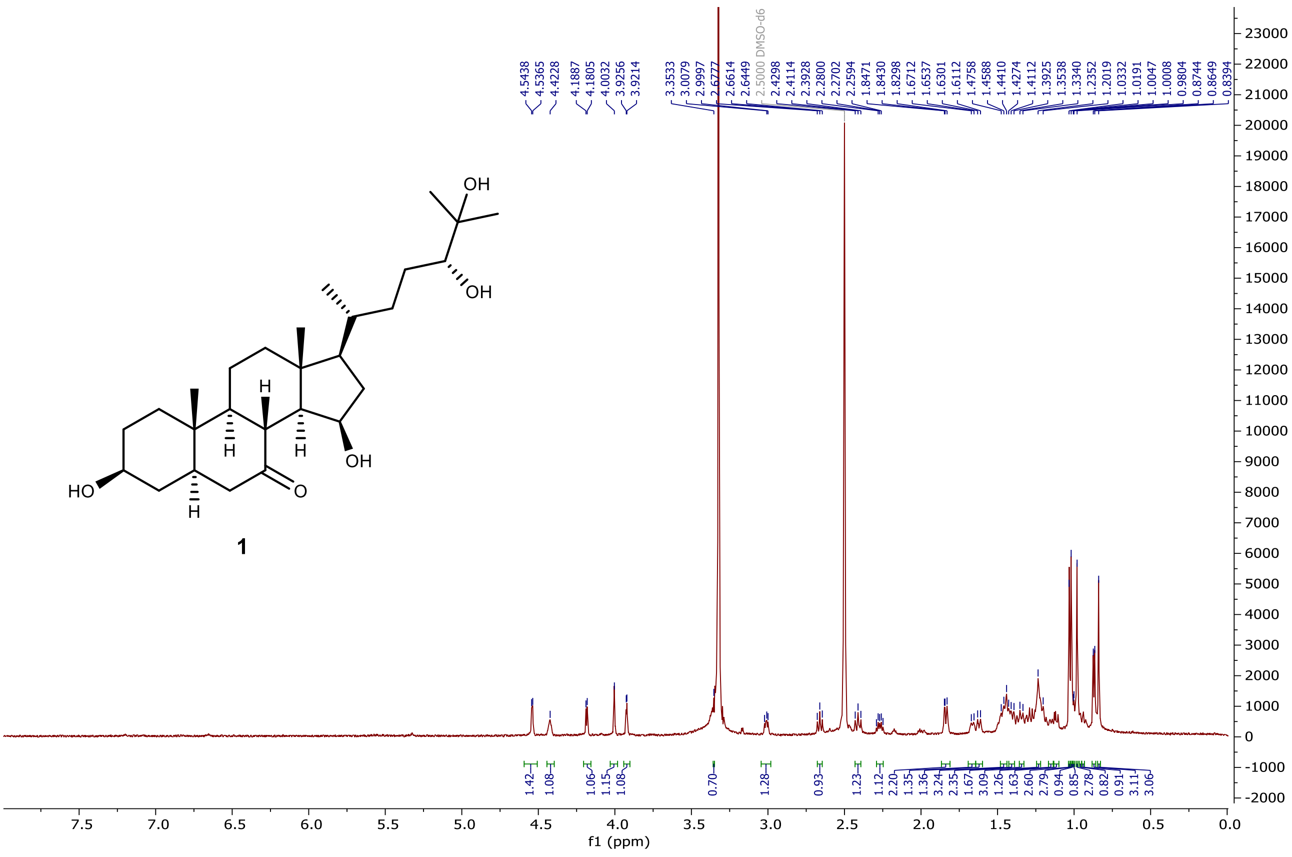

Figure S3. ^{1}H-NMR spectrum of nannosterol A (**1**) in DMSO-d_6 (700 MHz).

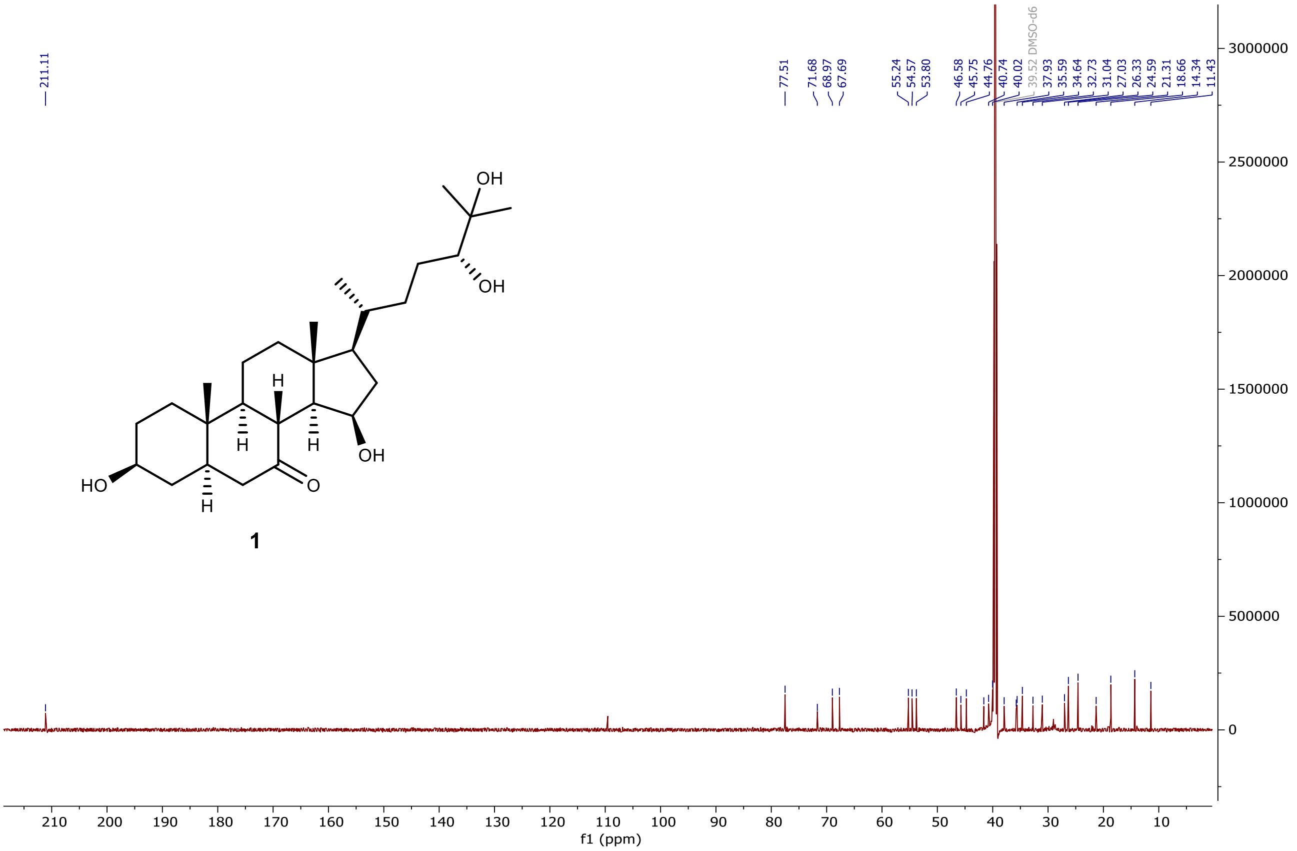

Figure S12. ^{13}C-NMR spectrum of nannosterol A (**1**) in DMSO-d_6. (175 MHz).

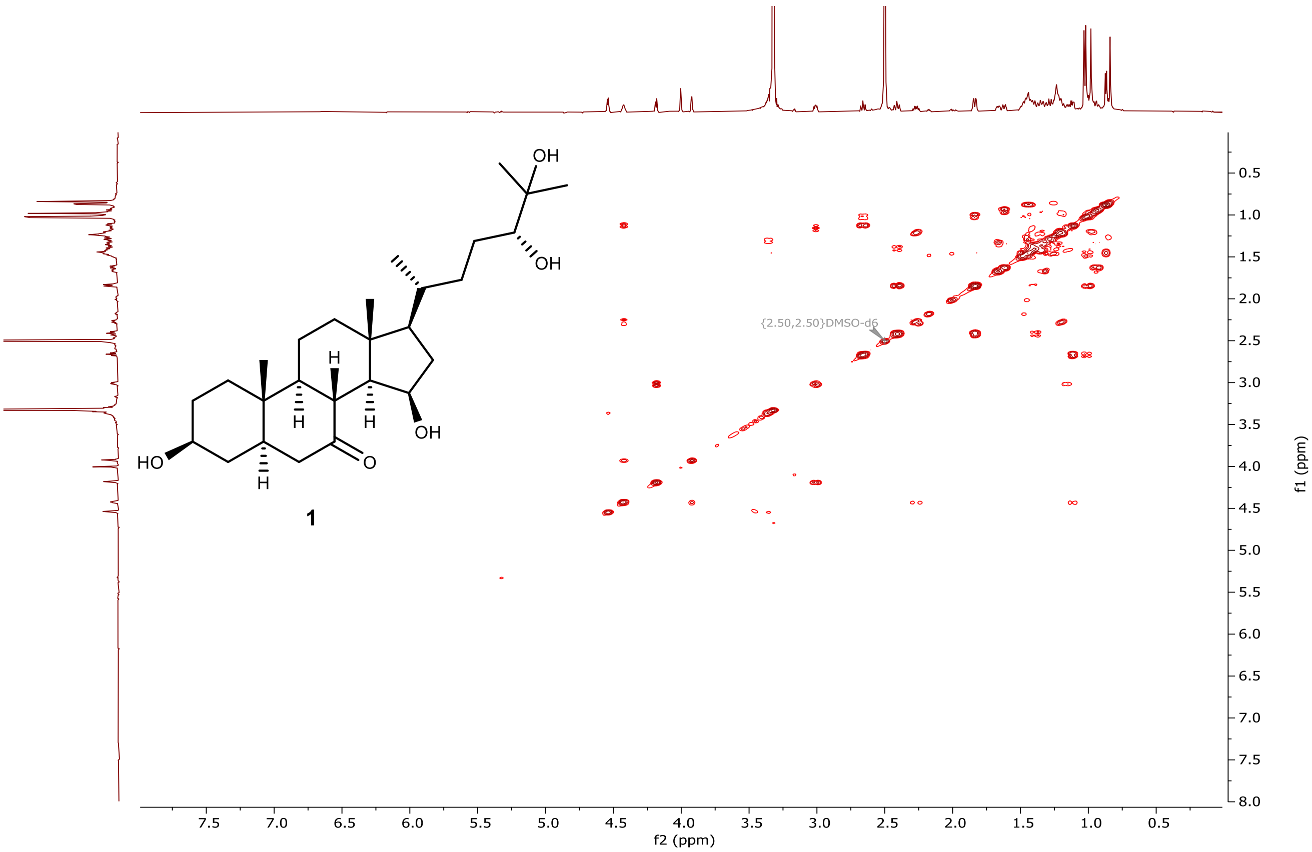

Figure S4. ^{1}H-^{1}H COSY spectrum of nannosterol A (**1**) in DMSO-d_6 (700 MHz).

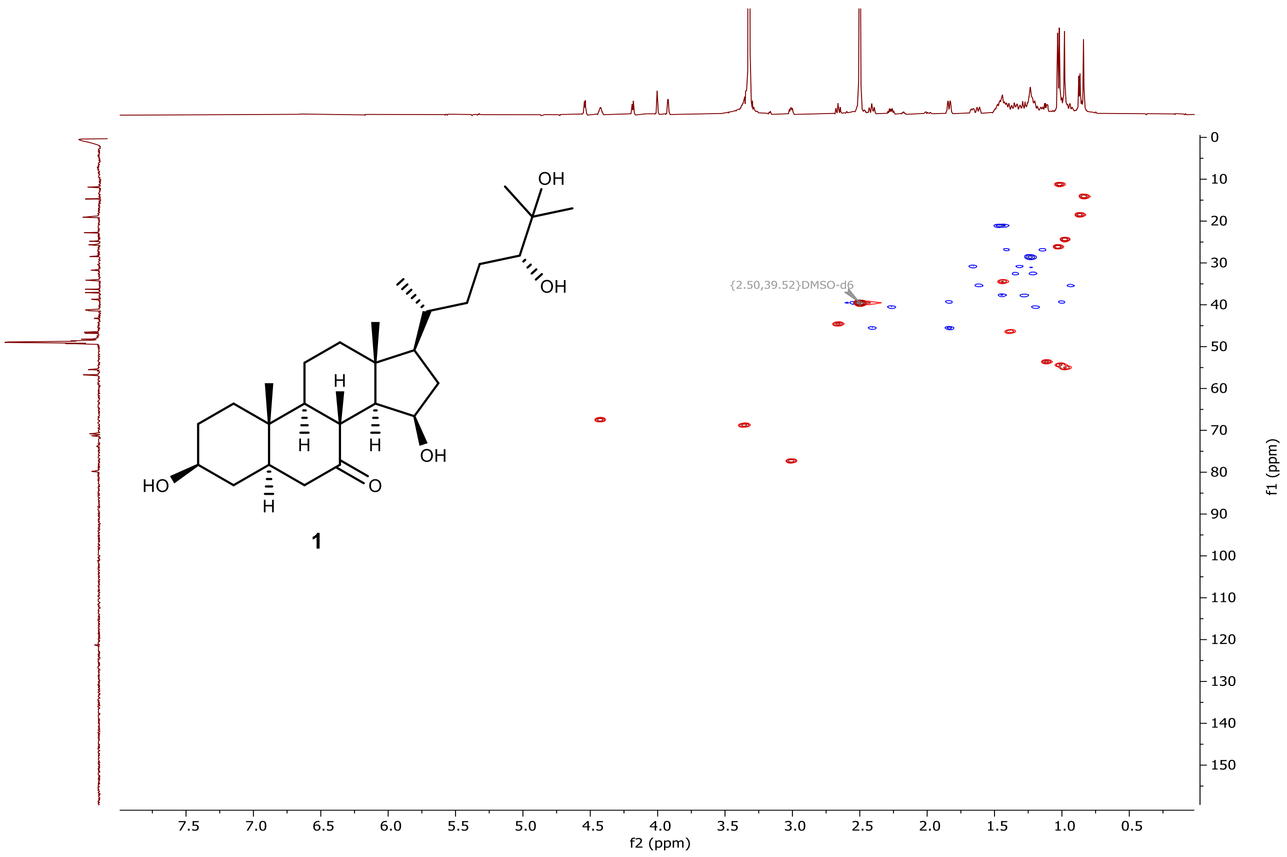

Figure S14. HSQC spectrum of nannosterol A (**1**) in DMSO-d_6 (700 MHz and 175 MHz).

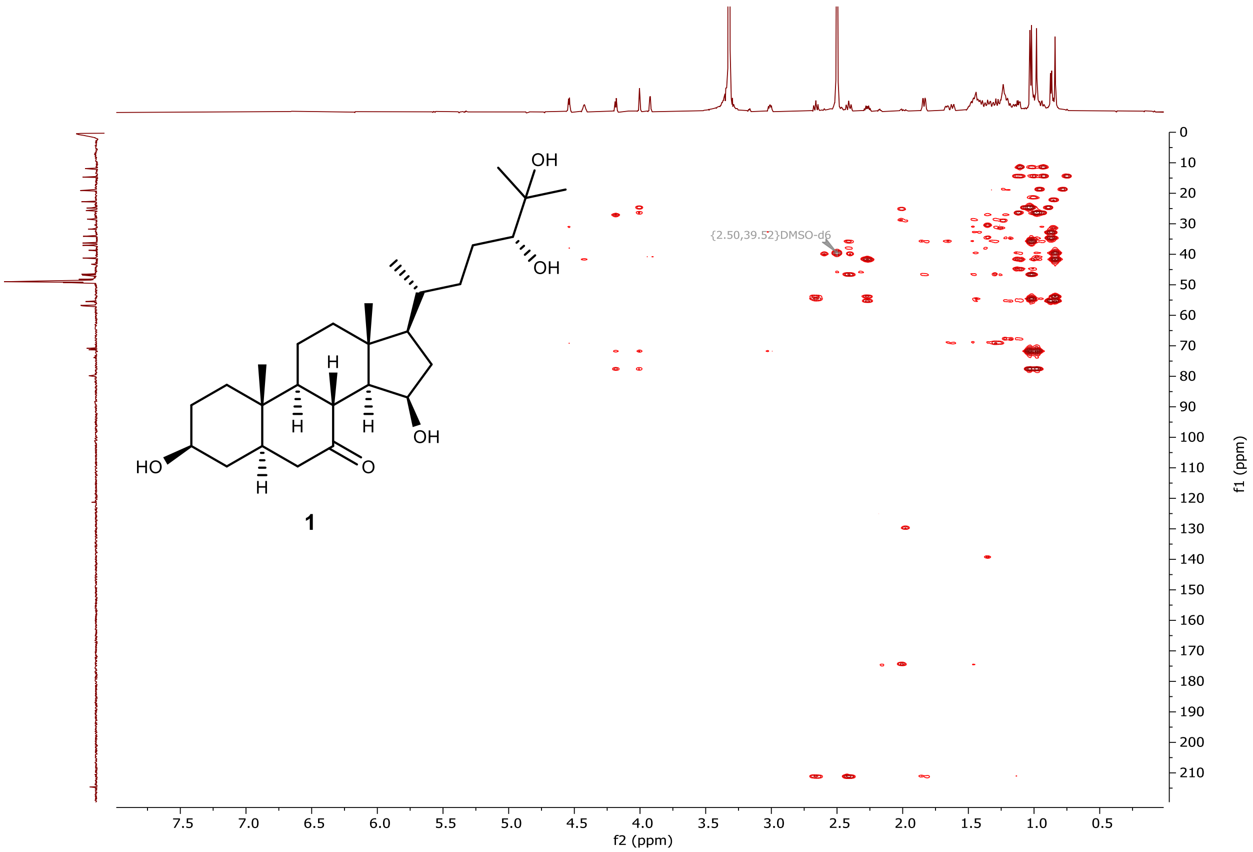

Figure S15. HMBC spectrum of nannosterol A (**1**) in DMSO-d_6 (700 MHz and 175 MHz).

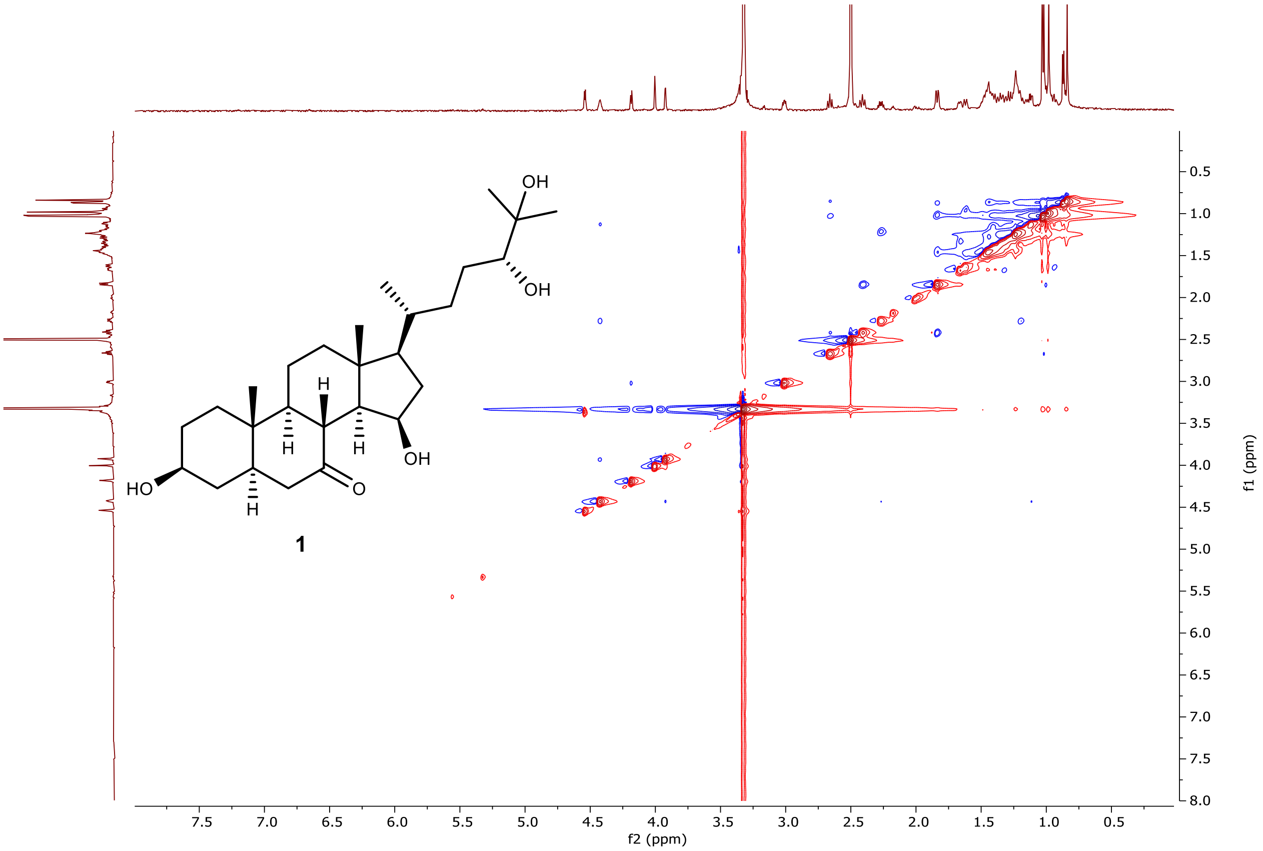

Figure S16. ROESY spectrum of nannosterol A (**1**) in DMSO-d_6 (700 MHz).

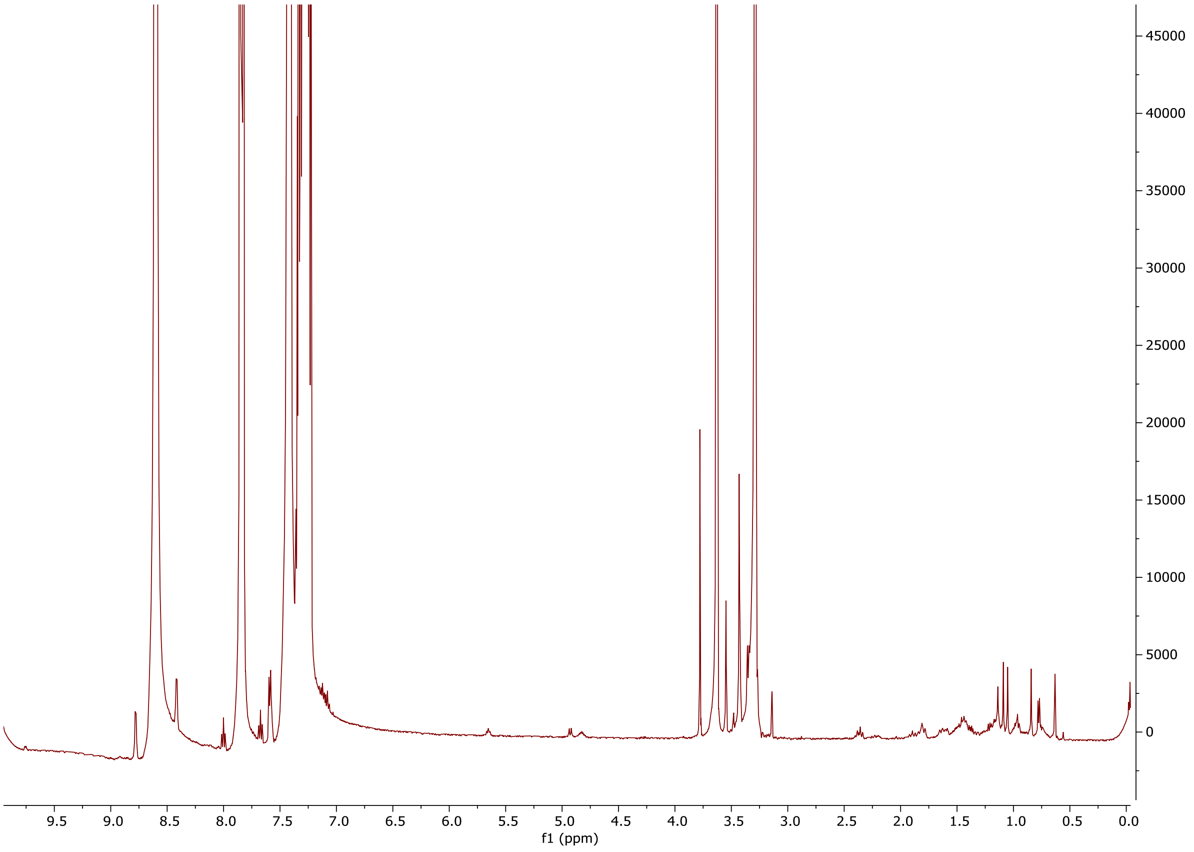

Figure S17. ^{1}H NMR spectrum of the (*S*)-MTPA ester derivative of nannosterol A (**1**) in $CDCl_3$ (500 MHz).

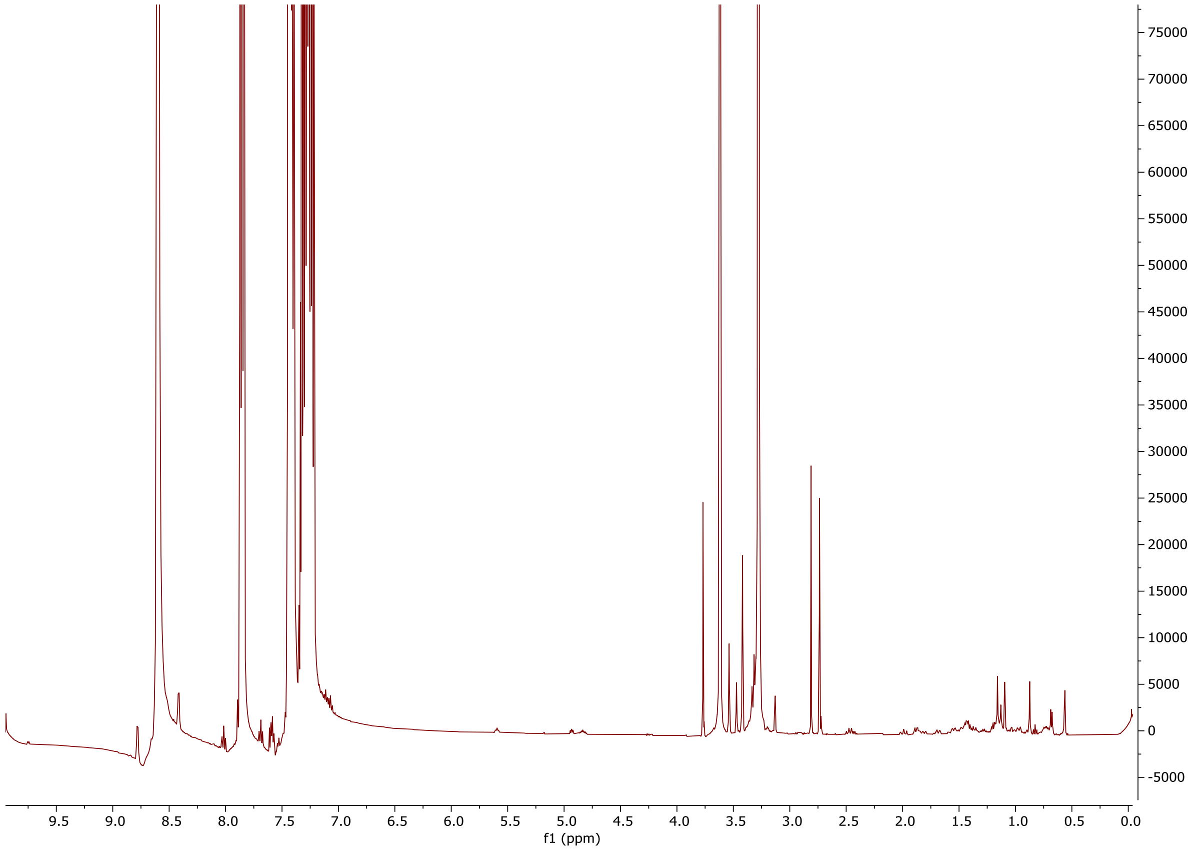

Figure S18. ^{1}H NMR spectrum of the (*R*)-MTPA ester derivative of nannosterol A (**1**) in $CDCl_3$ (500 MHz).

2.2 NMR spectra of Nannosterol B (**2**)

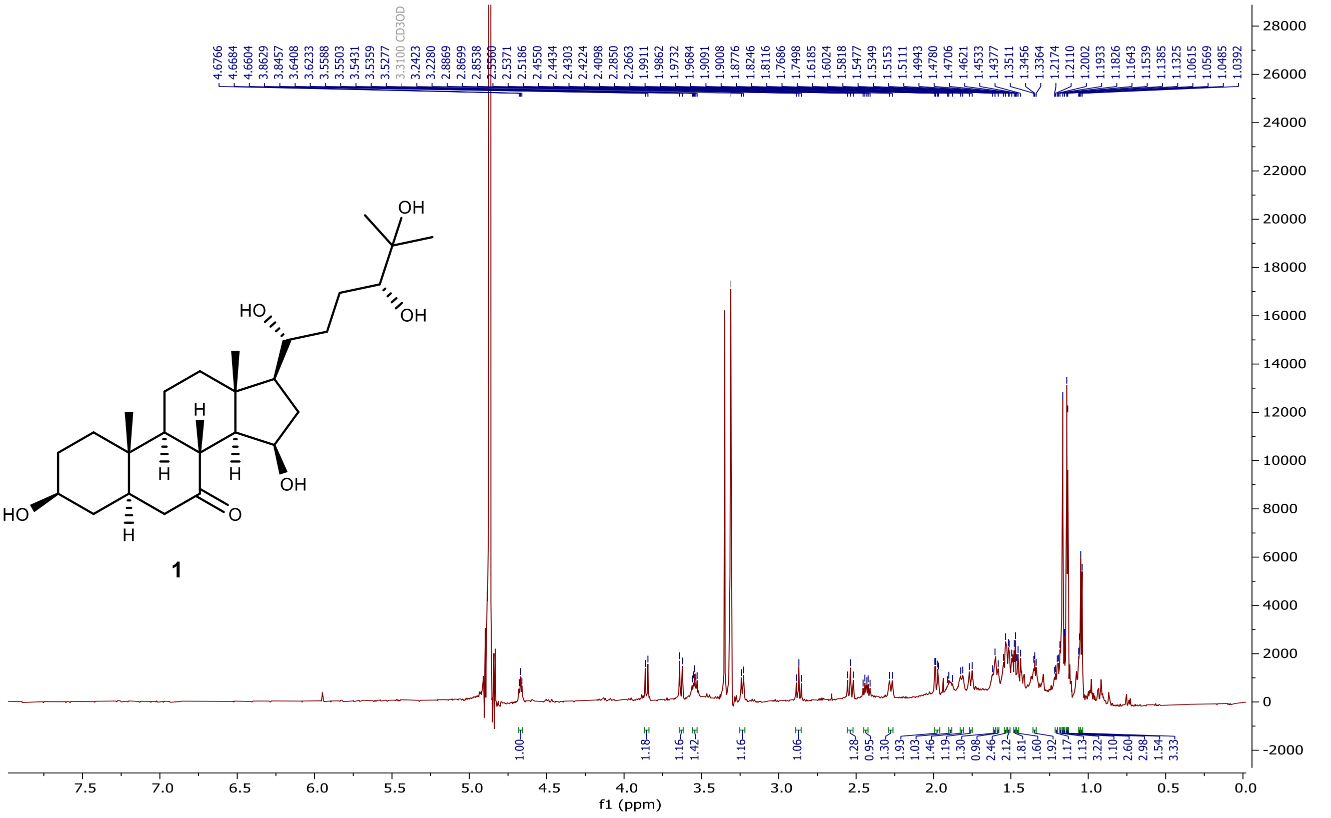

Figure S19. ^{1}H-NMR spectrum of nannosterol B (**2**) in CD_3OD (700 MHz).

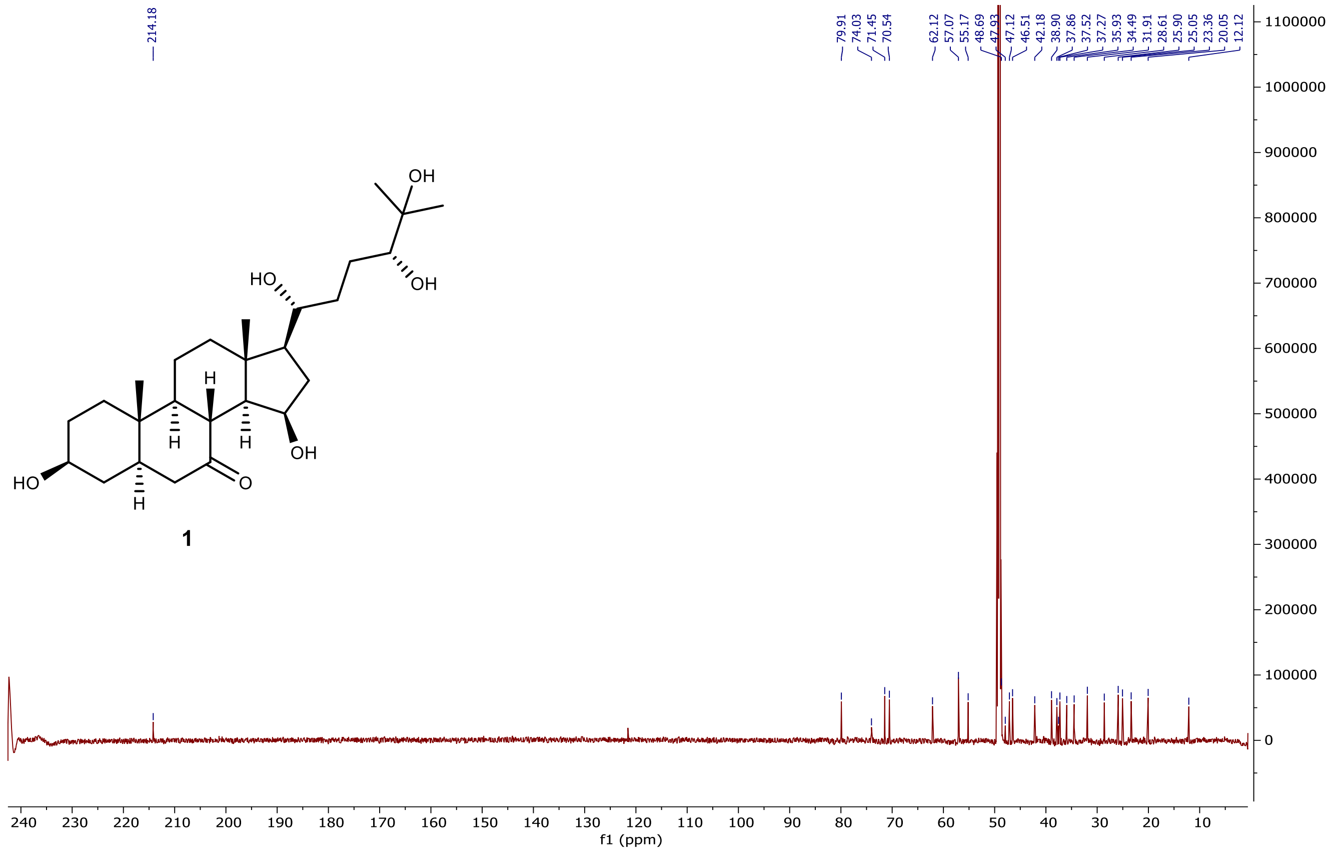

Figure S20. ^{13}C-NMR spectrum of nannosterol B (**2**) in CD_3OD (175 MHz).

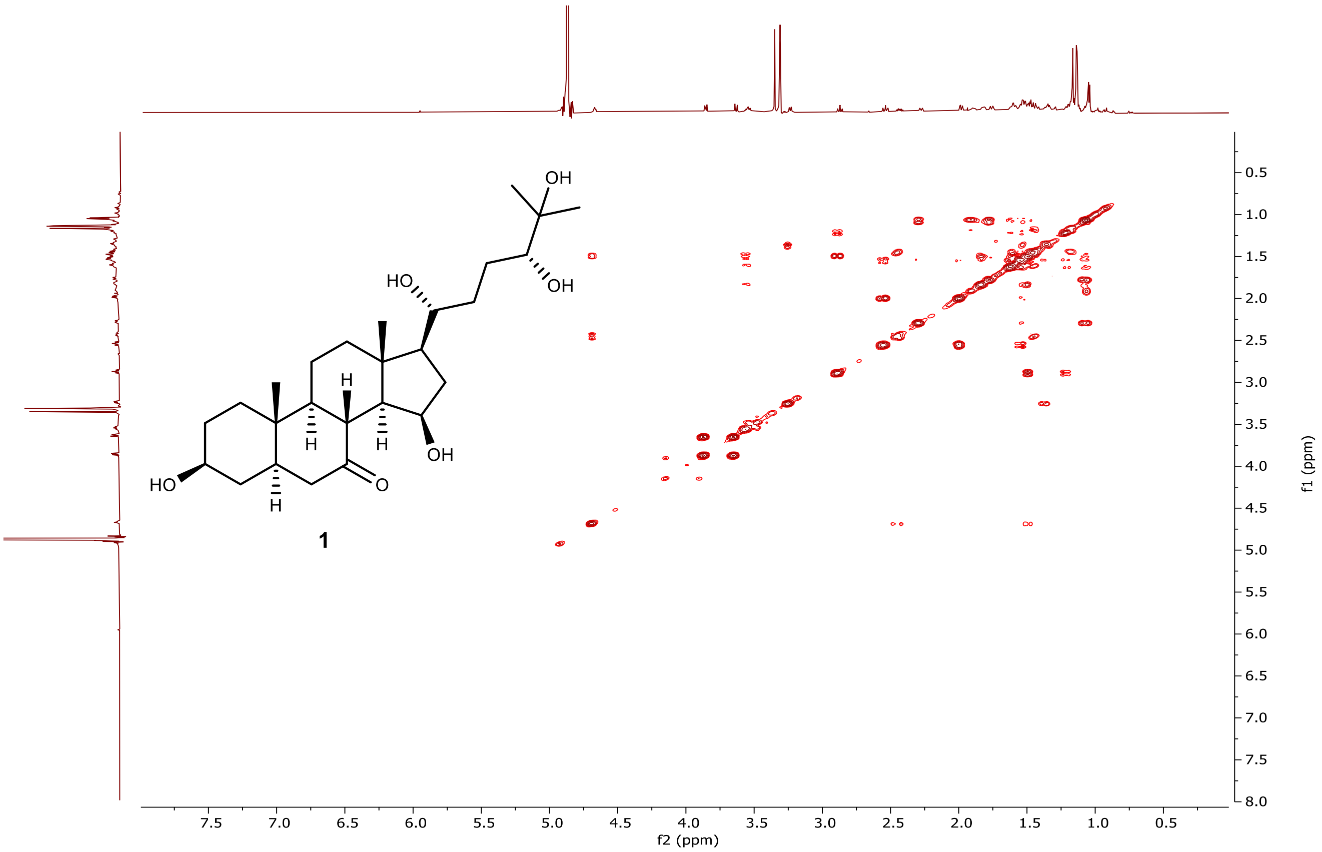

Figure S21. ${}^{1}H$-${}^{1}H$ COSY spectrum of nannosterol B (**2**) in CD_3OD (700 MHz).

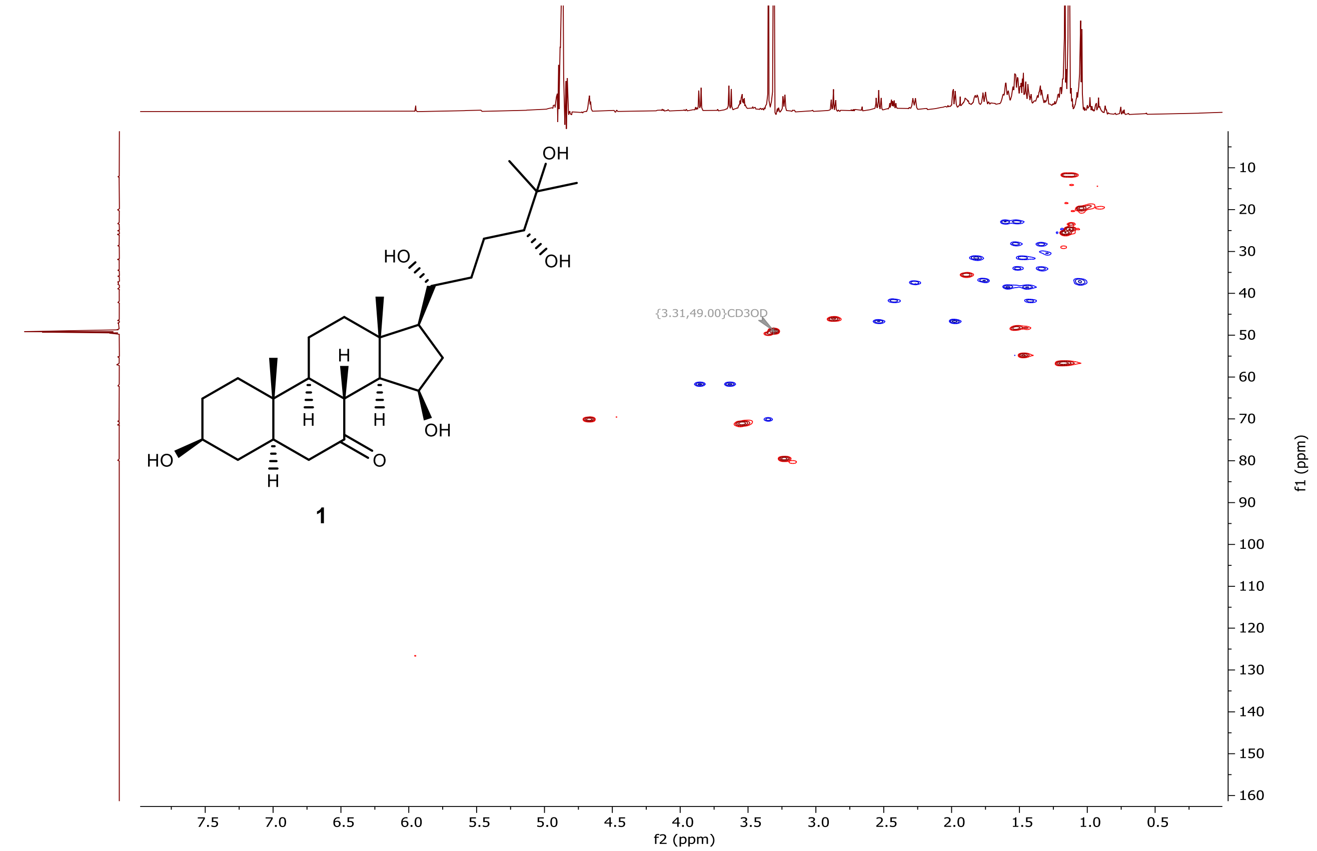

Figure S22. HSQC spectrum of nannosterol B (2) in CD_3OD (700 MHz and 175 MHz).

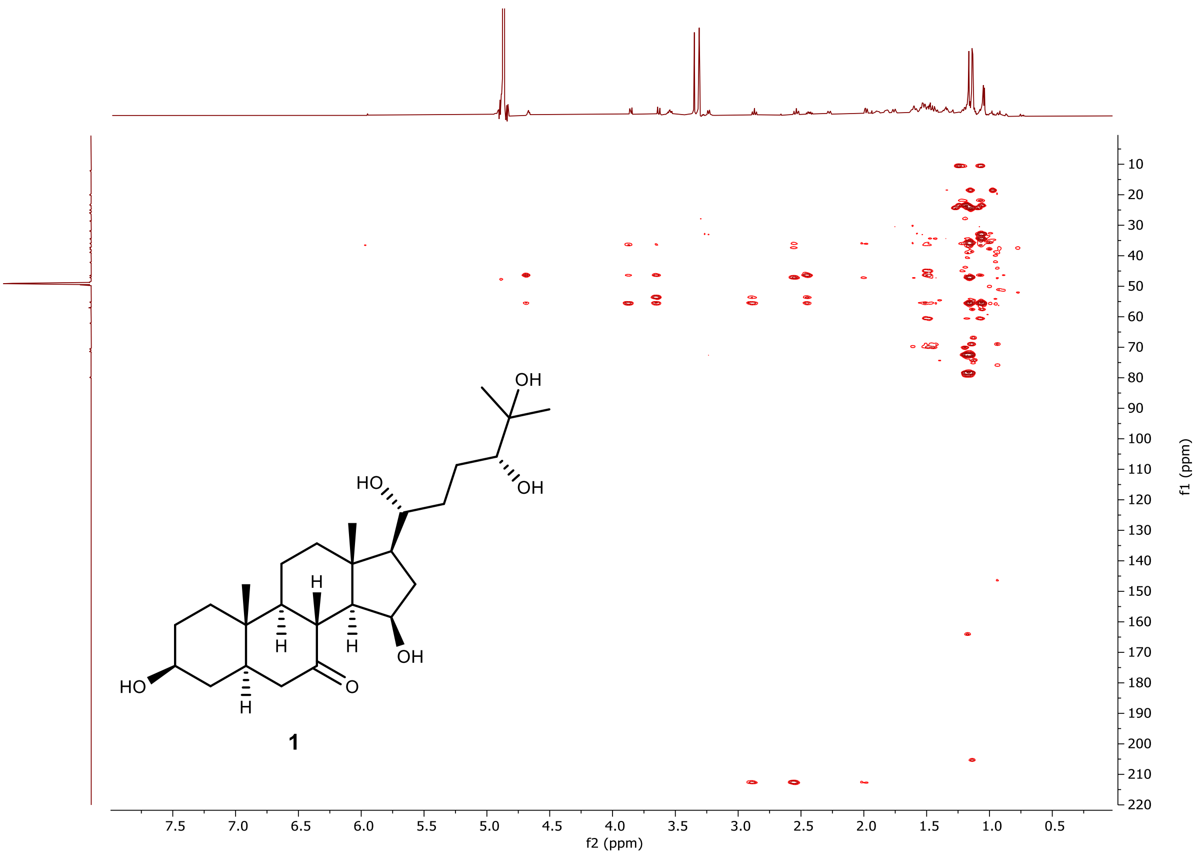

Figure S23. HMBC spectrum of nannosterol B (**2**) in CD_3OD (700 MHz and 175 MHz).

3. Crystallographic data of **1**

3.1 Refinement details for Nannosterol A (**1**)

All non H-atoms were located in the electron density maps and refined anisotropically. C-bound H atoms were placed in positions of optimized geometry and treated as riding atoms. Their isotropic displacement parameters were coupled to the corresponding carrier atoms by a factor of 1.2 (CH, CH_2) or 1.5 (CH_3). O bonded H atoms were located in the electron density maps. Their positional parameters were refined using isotropic displacement parameters, which were set at 1.5 times the Ueq value of the parent atoms. Restraints of 0.84 (0.01) Å were used for the O-H bond lengths.

Disorder: The condensed cyclohexane ring (C28A-C31A, C28B-C31B) including the methyl group (C46A, C46B) was split over two positions. Its occupancy factor refined to 0.81 for the major (A) product.

Table S1. Crystal data and structure refinement for **1** (Nannosterol A).

Identification code	sh4861_a	
CCDC-Nr.	2223627	
Empirical formula	C27 H46 O5	
Formula weight	450.64	
Temperature	133(2) K	
Wavelength	1.54178 Å	
Crystal system	Monoclinic	
Space group	$P2_1$	
Unit cell dimensions	a = 11.2919(8) Å	α= 90°.
	b = 19.6966(14) Å	β= 102.392(3)°.
	c = 11.5093(8) Å	γ = 90°.
Volume	2500.2(3) Å^3	
Z	4	
Density (calculated)	1.197 Mg/m^3	
Absorption coefficient	0.636 mm^{-1}	
F(000)	992	
Crystal size	0.160 x 0.140 x 0.080 mm^3	
Theta range for data collection	3.932 to 74.947°.	
Index ranges	-14<=h<=14, -23<=k<=24, -14<=l<=14	
Reflections collected	31466	
Independent reflections	9906 [R(int) = 0.0577]	
Completeness to theta = 67.679°	99.9 %	
Absorption correction	Semi-empirical from equivalents	
Max. and min. transmission	0.5871 and 0.4324	
Refinement method	Full-matrix least-squares on F^2	
Data / restraints / parameters	9906 / 307 / 691	
Goodness-of-fit on F^2	1.037	
Final R indices [I>2sigma(I)]	R1 = 0.0531, wR2 = 0.1454	
R indices (all data)	R1 = 0.0580, wR2 = 0.1491	
Absolute structure parameter	0.13(8)	
Extinction coefficient	n/a	
Largest diff. peak and hole	0.459 and -0.252 e.Å^{-3}	

4. Phylogenetic analysis of MNa10993

4.1 *Nannocystis* sp. MNa10993 16S ribosomal RNA nucleotide sequence

CTGGAGAGTTTGATCCTGGCTCAGAGCGAACGTTTGCGGCGGGCCTAACACATGCAAGTC
GAACGGGCTAGCAATAGTCAGTGGCGCACGGGTGCGTAACACGTAGGTAATCAACCCCT
CGGTTCGGGATAACGTTCTGAAAGGAGCGCTAATACCGGACGTGTCTTCGGGAGCTTCGG
CTCCTGTCGAGAAAGACCCGCAAGGGTTGCCGAGGGACGAGCCTGCGGCCCATCAGCTA
GTTGGCGAGGTAATAGCTCACCAAGGCGAAGACGGGTAGCTGGTCTGAGAGGATGATCA
GTCACACTGGAACTGAGACACGGTCCAGACTCCTACGGGAGGCAGCAGTGGGGAATATT
GCGCAATGGGCGAAAGCCTGACGCAGCCACGCCGCGTGAGCGATGAAGGCCTTCGGGTC
GTAAAGCTCTGTGGGGAGAGACGAAGAAAGCCTGTGAAGAGCAGGCCTTGACGGTATCT
CCTTAGCAAGCACCGGCTAACTCCGTGCCAGCAGCCGCGGTAATACGGAGGGTGCGAAC
GTTGCTCGGAATCATTGGGCGTAAAGCGCACGTAGGCGGCGGCGTAAGCGGGATGTGAA
AGCCCAGGGCTCAACCCTGGAAGTGCATCCCGAACTGCGTCGCTTGAATCTCGGAGGGGG
ACAGAGAATTCCCGGTGTAGAGGTGAAATTCGTAGATATCGGGAGGAATACCAGTGGCG
AAGGCGCTGTCCTGGACGAAGATTGACGCTGAGGTGCGAAAGCGTGGGGAGCAAACAGG
ATTAGATACCCTGGTAGTCCACGCTGTAAACGATGAGTGCTGGACGGTGGAGGATTTGAC
CCCTTCGCTGTCGAAGCTAACGCGTTAAGCACTCCGCCTGGGGAGTACGGTCGCAAGACT
AAAACTCAAAGGAATTGACGGGGGCCCGCACAAGCGGTGGAGCATGTGGTTTAATTCGA
CGCAACGCGCAGAACCTTACCTGGGTTAAATCCACTGGAACCTGGCTGAAAGGCTGGGGT
GCCCTTCGGGGAGCCGGTGAGAAGGTGCTGCATGGCTGTCGTCAGCTCGTGTCGTGAGAT
GTTGGGTTAAGTCCCGCAACGAGCGCAACCCCTATCGCCAGTTGCCACCATTGAGTTGGG
AACTCTGGCGAGACTGCCGGTCTAAACCGGAGGAAGGTGGGGACGACGTCAAGTCCTCA
TGGCCCTCATGCCCAGGGCTACACACGTGCTACAATGGCTGGTACAAAGAGCCGCAAGCC
CGCGAGGGTGAGCAAATCTCAAAAAACCAGTCTCAGTTCGGATTGCAGTCTGCAACTCGA
CTGCATGAAGCTGGAATCGCTAGTAATCGGAGATCAGCACGCTCCGGTGAATACGTTCCC
GGGCCTTGTACACACCGCCCGTCACACCATGGGAGTCGGCTGCTCCAGAAGTAGGAACCT
CAACCGCAAGGAAAGGCCCTACCAAGGAGCGGTCGGTGACTGGGGTGAAGTCGTAACAA
GGTAGCCGTAGGGGAACCTGCGGCTGGATCACCTCCTTT

4.2 Phylogenetic tree

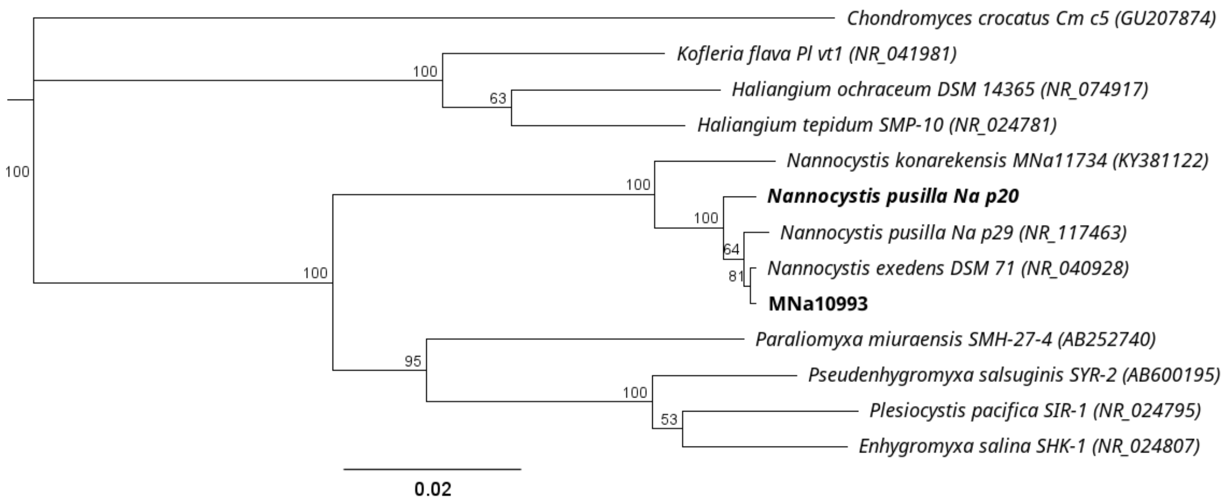

Figure S24. Neighbor-joining tree inferred based on 16S rRNA gene sequence showing the position of myxobacterial strain MNa10993 in *Nannocystineae* suborder and its closely related *Nannocystis* species. The sequence of *Chondromyces crocatus* strain Cm c5^{T} was used to root the tree. Bar, 0.02 nucleotide substitution per site.

5. Metabolome investigation

5.1 Identification of the genome-sequenced alternative producer of **1–2** *Nannocystis pusilla* Na p20 (DSM 53165)

Table S2. Identified myxobacterial producers of Nannosterols **1–2**.

Strain	Nannosterol A **1**	Nannosterol B **2**
Nannocystis sp. (MNa10993) (original isolation of **1–2**)	+	+
Nannocystis pusilla Na p20 (DSM 53165, MNa2747)	+	+
Nannocystis excedens Na e67 (DSM 53053, MNa2943)	+	−
Nannocystis excedens Na e711 (MNa2925)	+	−
Nannocystis unclassified SBNa024 (MNa10659)	+	+
Nannocystis unclassified SBNa035 (MNa10741)	+	+
Nannocystis unclassified SBNa023 (MNa10658)	+	+
Nannocystis exedens Na e603 (MNa217)	+	−
Nannocystis unclassified SBNa026 (MNa10661)	+	+
Nannocystis unclassified SBNa027 (MNa10662)	+	+
Nannocystis unclassified SBNa032 MNa10667	+	+
Nannocystis unclassified SBNa025 MNa10660	+	+
Nannocystis execedens Na c33 (DSM 25994, MNa3377)	+	+
Nannocystis unclassified SBNa021 (MNa10656)	+	+
Nannocystis unclassified (MNa10991)	+	+
Nannocystis pusilla Na p19 (SBNa2749)	+	+

(+) detected; (–) not detected

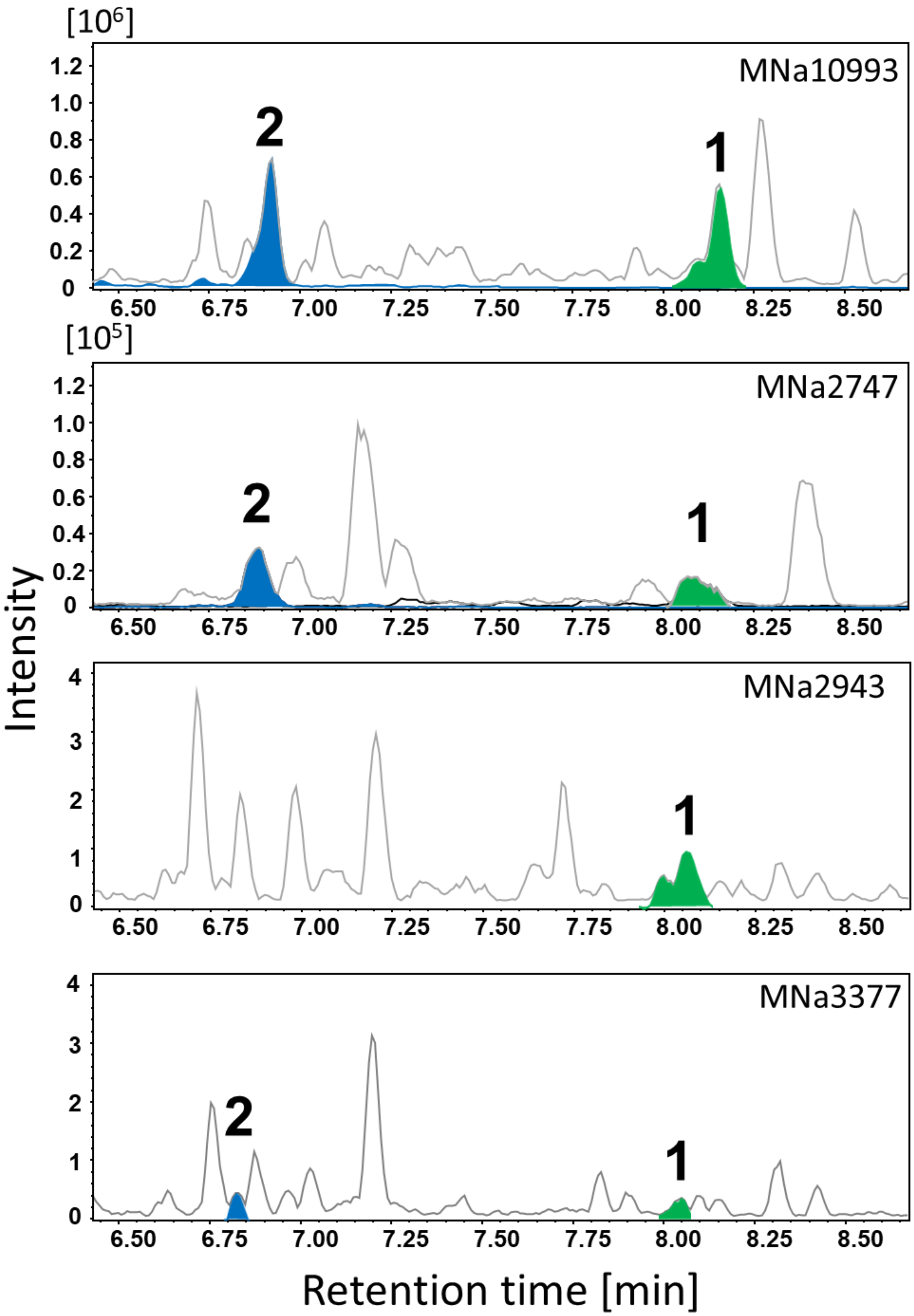

Figure S25. High performance liquid chromatography–mass spectrometry extracted ion chromatograms (HPLC–MS EIC) of **1** ($[M + H]^+$ 451.3397 *m/z*, green), **2** ($[M + H]^+$ 467.3341 *m/z*, blue) from the myxobacterial crude extracts of MNa10993, MNa2747, MNa2943 and MNa3377.

6. Genetic and biosynthetic investigations

6.1 Identified Terpene BGCs in *Nannocystis pusilla* Na p20 (DSM 53165).

Table S3. *In silico* analysis of the genome-sequenced alternative producer of **1–2** *Nannocystis pusilla* Na p20 (DSM 53165) with the antibiotics and secondary metabolite analysis shell (antiSMASH) (NCBI Reference Sequence: NZ_JAIRAU000000000.1). Detection strictness: relaxed; Used extra features; KnownClusterBlast, ClusterBlast SubClusterBlast, MIBiG cluster comparison, ActiveSiteFinder, RREFinder, Cluster Pfam analysis, Pfam-based GO term annotation, TIGRFam analysis. Accessed on 18.10.2022.

No.	Contig ID	Location From – To	Size (bp)	Proposed function
1	JAIRAU010000005.1	55609–77490	20882	---
2	JAIRAU010000008.1	32661–53672	21011	---
3	JAIRAU010000017.1	1–13378	13378	Geosmin production
4	JAIRAU010000027.1	334488–355456	20969	---
5	JAIRAU010000028.1	474608–495594	20987	--
6	JAIRAU010000040.1	115829–137823	21995	Contains OSC gene homolog for lanosterol biosynthesis
7	JAIRAU010000048.1	29042–50028	20987	Carotenoid biosynthesis
8	JAIRAU010000049.1	148433–169611	21179	--
9	JAIRAU010000050.1	25178–46089	20912	--
10	JAIRAU010000058.1	1–12007	12007	Harbors gene homologs of *hpnCDE* for squalene biosynthesis

6.2 Steroid biosynthesis genes in *Nannocystis pusilla* Na p20 (DSM 53165)

Table S4. Previously identified sterol biosynthesis genes in bacterial genomes and identified gene homologs in the genome-sequenced alternative producer of **1–2** *Nannocystis pusilla* Na p20 (DSM 53165).

**Gene**	**UniProt**	***Nannocystis pusilla* Na p20 (DSM53165)[a,b]**
**Terpene Cyclases**		
SHC	P33247	1st hit: JAIRAU010000040 / K7C98_29055
		2nd hit: JAIRAU010000057 / K7C98_41465
STC	Q24FB1	1st hit: JAIRAU010000040 / K7C98_29055
		2nd hit: JAIRAU010000057 / K7C98_41465
**Phytoene Synthase (to identifiy carotenoid synthases)**		
CrtM	P37269	JAIRAU010000048 / K7C98_35210
**Steroid biosynthesis genes stage 1**		
**HMG-CoA reductase pathway genes**		
HMGS	P54868	JAIRAU010000028.1/ K7C98_21665
HMGR	P13702	---
MVK	Q03426	JAIRAU010000009/ K7C98_11205
PMVK	Q15126	------
DPMD	P53602	JAIRAU010000009/ K7C98_11200
IPPI	Q9KWG2	JAIRAU010000009/ K7C98_11210
**MEP Pathway**		
DXR	P45568	-------
CMK	P62615	-------
**FPPS Biosynthesis**		
FPPS (*E. coli)*	P14324	1) JAIRAU010000039 / K7C98_27790
		2) JAIRAU010000023 / K7C98_17230
FPPS (human)	P22939	1) JAIRAU010000057/ K7C98_40830
		2) JAIRAU010000044 / K7C98_31500
**Squalene formation**		
**HpnCDE pathway (three-step)**		
HpnC	A6G141	JAIRAU010000058 / K7C98_41530
HpnD	A6G142	JAIRAU010000058 / K7C98_41535
HpnE	Q5NP65	JAIRAU010000058 / K7C98_41540
**Squalene synthase single-step pathway**		
SQS	P37268	-----
**Steroid biosynthesis genes stage 2**		
SQMO	Q14534	JAIRAU010000057/ K7C98_41470
AltSQMO	B7FXW1	JAIRAU010000001/ K7C98_01780
OSC	P48449	JAIRAU010000040 / K7C98_29055
SC	CCE24201	JAIRAU010000040/ K7C98_29055
CYP51	Q16850	JAIRAU010000031 / K7C98_24805
ERG24	Q14739	JAIRAU010000012 / K7C98_12900
ERG25	Q15800	JAIRAU010000001 / K7C98_05955
ERG26	Q15738	JAIRAU010000001/ K7C98_01345
ERG27	P38694	Numerous homologs
ERG28	Q9UKR5	----
SdmA	Q60A55	JAIRAU010000031 / K7C98_24795
SdmB	Q60A54	JAIRAU010000002 / K7C98_07170
SdmC	A0A0C2CRB7	JAIRAU010000045 / K7C98_32865
CPI1	Q9M643	JAIRAU010000056 / K7C98_39540
SMT	P25087	JAIRAU010000057 / K7C98_41210

ERG2	P32352	---
EBP (ERG2 homolog)	Q15125	JAIRAU010000044 / K7C98_32095
ERG3	O75845	JAIRAU010000001 / K7C98_05955
DHCR7	Q9UBM7	JAIRAU010000012 / K7C98_12900
ERG4	P25340	JAIRAU010000012 / K7C98_12900
DHCR24 (ERG4 homolog)	Q15392	JAIRAU010000051 / K7C98_36740
CYP61	P54781	JAIRAU010000029 / K7C98_23820
**Putative Tailoring genes**		
**C18-Hydroxylase**		
CYP11B3	P30100	JAIRAU010000029 / K7C98_23820
CYP11B2	P19099	JAIRAU010000045 / K7C98_32855
**C25-Hydroxylation**		
Cholesterol 25-Hydroxylase	O95992	---
C25-Dehydrogenase	H9NN89	---
**Putative genes required for the biosynthesis of 1 and 2 (according to Figure 7)**		
HMGS	P54868	JAIRAU010000028.1/ K7C98_21665
HMGR	P13702	---
MVK	Q03426	JAIRAU010000009/ K7C98_11205
PMVK	Q15126	------
DPMD	P53602	JAIRAU010000009/ K7C98_11200
IPPI	Q9KWG2	JAIRAU010000009/ K7C98_11210
HpnD	A6G142	JAIRAU010000058 / K7C98_41535
HpnC	A6G141	JAIRAU010000058 / K7C98_41530
HpnE	Q5NP65	JAIRAU010000058 / K7C98_41540
SQMO	Q14534	JAIRAU010000057/ K7C98_41470
OSC	P48449	JAIRAU010000040 / K7C98_29055
ERG24	Q14739	JAIRAU010000012 / K7C98_12900
SdmA	Q60A55	JAIRAU010000031 / K7C98_24795
SdmB	Q60A54	JAIRAU010000002 / K7C98_07170
SdmC	A0A0C2CRB7	JAIRAU010000045 / K7C98_32865
ERG25	Q15800	JAIRAU010000001 / K7C98_05955
ERG26	Q15738	JAIRAU010000001/ K7C98_01345
ERG27	P38694	Numerous homologs
EBP	Q15125	JAIRAU010000044 / K7C98_32095
ERG3	O75845	JAIRAU010000001 / K7C98_05955
DHCR7	Q9UBM7	JAIRAU010000012 / K7C98_12900
DHCR24 (ERG4 homolog)	Q15392	JAIRAU010000051 / K7C98_36740

SHC: squalene-hopene cyclase; STC: squalene tetrahymanol cyclase; CrtM:15-cis phytoene synthase; HMGS: 3-hydroxy-3-methylglutaryl-coenzyme A synthase; HMGR: 3-hydroxy-3-methylglutaryl-coenzyme A reductase; MVK: mevalonate kinase; PMVK: phosphomevalonate kinase; DPMD: diphosphomevalonate decarboxylase; DXR: 1-deoxy-D-xylulose-5-phosphate reductoisomerase; CMK: 4-diphosphocytidyl-2C-methyl-D-erythritol kinase; FPPS: farnesyl pyro/diphosphate synthase; HpnC: hydroxysqualene synthase; HpnD: presqualene diphosphate synthase; HpnE: hydroxysqualene dehydroxylase; SQS: squalene synthase; SQMO: squalene monooxygenase; AltSQMO: alternative squalene epoxidase/monooxygenase; OSC: oxidosqualene cyclase; SC: Squalene cyclase; CYP51: lanosterol 14-alpha demethylase/C-14 demethylase (ERG11 homolog); ERG24: delta(14)-sterol reductase; ERG25: C-4 methylsterol oxidase; ERG26: sterol-4-alpha-carboxylate 3-dehydrogenase; ERG27: C-3 keto sterol reductase; ERG28: ergosterol biosynthetic protein 28; SdmA: sterol demethylase protein A/4beta-methylsterol monooxygenase; SdmB: sterol demethylase protein B/ 3beta-hydroxysteroid-4beta-carboxylate 3-dehydrogenase; CPI1: Cycloeucalenol cycloisomerase; SMT: sterol 24-C-methyltransferase ERG6; ERG2: C-8 sterol isomerase; EBP: 3-beta-hydroxysteroid-*d*elta(8),*d*elta(7)-isomerase; ERG3: Delta(7)-sterol 5(6)-desaturase/ C-5 desaturase; DHCR7: 7-dehydrocholesterol reductase; ERG4: Delta(24(24(1)))-sterol reductase; DHCR24: Delta(24)-sterol reductase; CYP61: C-22 sterol desaturase; CYP11B3: Cytochrome P450 11B3; CYP11B2: Cytochrome P450 11B2.

6.3 Sequence alignments and Phyre2 structure

OSC alignment to discriminate between lanosterol and cycloartenol production

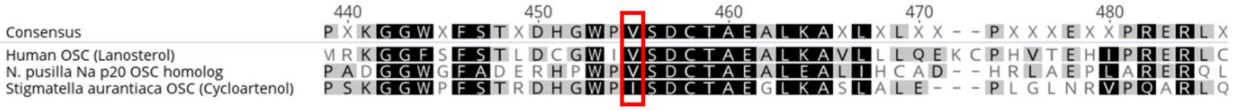

Figure S26. Amino acid alignment of human OSC (UniProt: P48449), the identified myxobacterial OSC homologs from *Nannocystis pusilla* Na p20 (K7C98_29055) and from *Stigmatella aurantiaca* (UniProt: Q7WZD1). The residue **453 (numbering according to human OSC)** indicates lanosterol or cycloartenol production; the identified OSC in *Nannocystis pusilla* Na p20 shows a V453, which indicates lanosterol production.

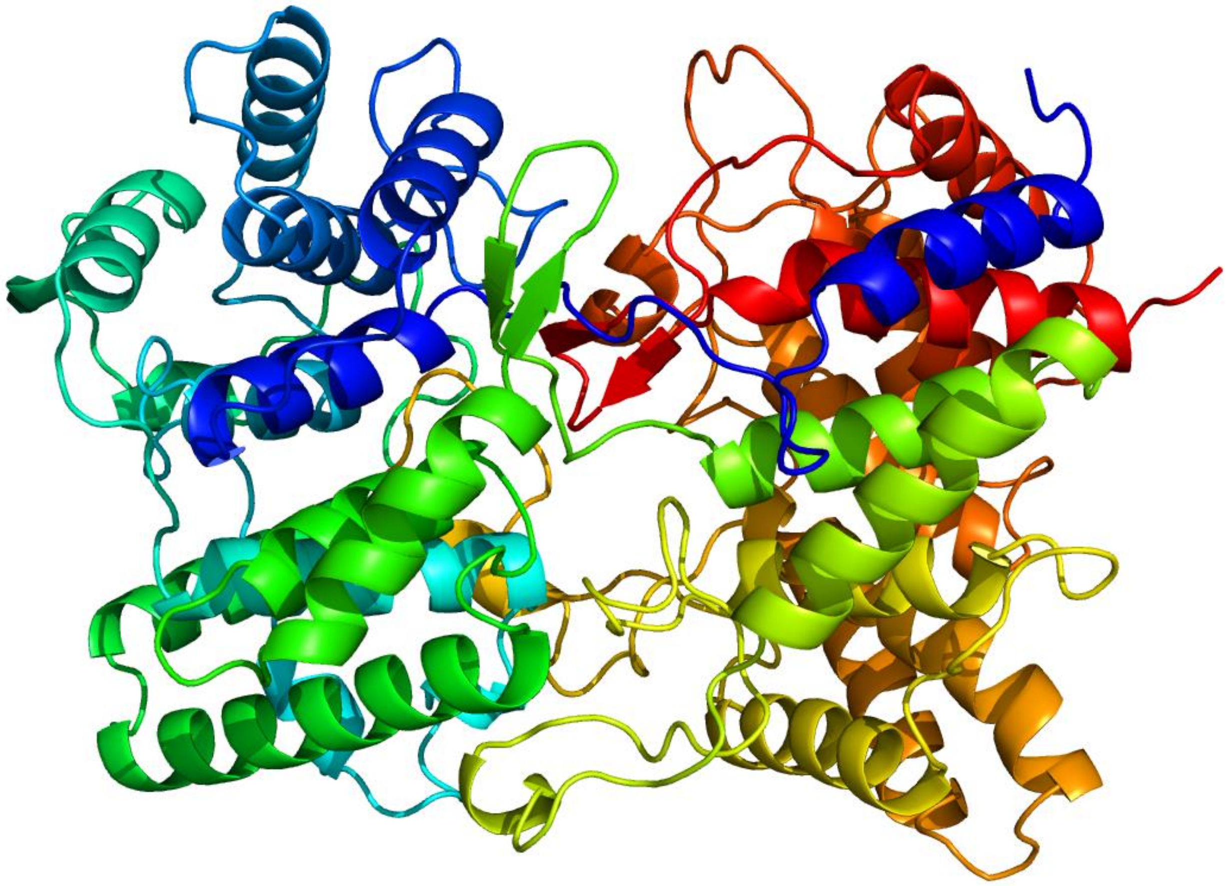

Figure S27. Phyre2 structure homology model of the encoded myxobacterial OSC homolog from *Nannocystis pusilla* Na p20 (K7C98_29055) (model is based on the template c1w6kA; figure colored by rainbow $N \rightarrow C$ terminus).

7. References

1 Dong, C., Flecks, S., Unversucht, S., Haupt, C., van Pée, K.-H., Naismith, J.H. (2005) Tryptophan 7-halogenase (PrnA) structure suggests a mechanism for regioselective chlorination. *Science (New York, N.Y.)*, **309** (5744), 2216–2219.

2 Henikoff, S. and Henikoff, J.G. (1992) Amino acid substitution matrices from protein blocks. *PNAS*, **89** (22), 10915–10919.

3 D'Agostino, P.M., Seel, C.J., Gulder, T., Gulder, T. (2021) *(Bio-)Synthesis of the Aquatic Phytotoxin Cyanobacterin – A Paradigm for Furanolide Core Structure Assembly.*

4 Neumann, P., Weidner, A., Pech, A., Stubbs, M.T., Tittmann, K. (2008) Structural basis for membrane binding and catalytic activation of the peripheral membrane enzyme pyruvate oxidase from Escherichia coli. *PNAS*, **105** (45), 17390–17395.

5 Lee, W.C., Cheon, D., Kim, Y. (2019) *Crystal structure of KAS III from Propionibacterium acnes.*

6 Hou, J., Chruszcz, M., Zheng, H., Cooper, D.R., Chordia, M.D., Zimmerman, M.D., Anderson, W.F., Minor, W. (2014) *Beta-ketoacyl-(acyl carrier protein) synthase III-2 (FabH2) from Vibrio cholerae soaked with Acetyl-CoA.*
